# Potential of Antimicrobial Peptide Synergies for Combating Infectious Diseases in Aquaculture: A Review

**DOI:** 10.3390/ani16121774

**Published:** 2026-06-08

**Authors:** Yanqi Zhang, Ning-Xi Tan, Xuan Ge, Jia-Feng Cao, Ya-Zhen Hu, Jiong Chen

**Affiliations:** 1 State Key Laboratory for Quality and Safety of Agro-Products, Ningbo University, Ningbo 315211, China; zhangyanqi@nbu.edu.cn (Y.Z.); caojiafeng@nbu.edu.cn (J.-F.C.); 2 Laboratory of Biochemistry and Molecular Biology, School of Marine Sciences, Ningbo University, Ningbo 315832, China; 246000930@nbu.edu.cn (N.-X.T.); 15267844534@163.com (X.G.); 3 Key Laboratory of Aquacultural Biotechnology, Ministry of Education, School of Marine Sciences, Ningbo University, Ningbo 315832, China

**Keywords:** antimicrobial peptides (AMPs), antibiotic resistance, synergistic therapy, synergistic mechanism, biomedical & agricultural applications

## Abstract

The increasing emergence of antimicrobial resistance in aquaculture has highlighted the urgent need for alternatives to conventional antibiotics. Antimicrobial peptides (AMPs) are promising candidates because of their broad-spectrum antimicrobial activity, multitarget mechanisms, and low propensity to induce resistance. However, their application is limited by poor stability, rapid degradation, and low bioavailability. This review summarizes recent advances in AMP-based synergistic strategies for controlling infectious diseases in aquaculture, including AMP-antibiotic, AMP-polysaccharide, AMP-herbal extract, and AMP-AMP combinations. Current evidence indicates that these strategies can enhance antimicrobial efficacy, reduce effective dosages, delay resistance development, improve AMP stability and delivery, and strengthen host immune responses. The underlying mechanisms involve membrane permeabilization, biofilm disruption, metabolic interference, controlled release, targeted delivery, and immunomodulation. Among these approaches, AMP-antibiotic combinations improve the effectiveness of existing antibiotics, while polysaccharide-based systems enhance peptide stability and bioavailability. AMP-herbal extract and AMP-AMP combinations further broaden antimicrobial activity through complementary mechanisms. Overall, AMP-based synergistic strategies represent promising alternatives for reducing antibiotic dependence in aquaculture. Future studies should focus on mechanistic validation, delivery optimization, field-scale evaluation, and regulatory development to facilitate their practical application.

## Simple Summary

The overuse of antibiotics in aquaculture has led to the serious problem of drug-resistant bacteria, threatening both animal health and food safety. Antimicrobial peptides (AMPs) are natural molecules that can kill a wide range of pathogenic bacteria without easily causing resistance. However, the application of AMPs in aquaculture environments remains challenging because they can break down quickly and lose effectiveness. This review examines different ways to combine AMPs with other substances, such as traditional antibiotics, plant extracts, or other types of AMPs. These combined strategies are generally more effective than AMP monotherapy, and they destroy bacterial membranes more effectively, disrupt protective biofilms, and reduce the likelihood of resistance development. The article highlights how these approaches can help reduce our dependence on antibiotics in fish and shellfish farming, supporting healthier animals and more sustainable aquaculture practices.

## 1. Introduction

Aquaculture is a cornerstone of global food security, supplying more than 15% of animal protein for human consumption and supporting livelihoods in coastal and inland communities [1]. However, the intensification of aquaculture systems, which are characterized by high stocking densities and simplified ecosystems, has exacerbated bacterial disease outbreaks, posing a critical threat to industry sustainability [2,3]. Pathogens such as *Streptococcus agalactiae* (causing streptococcosis in tilapia), *Aeromonas hydrophila* (gill rot and septicemia in freshwater fish), and *Vibrio* spp. (early mortality syndrome, EMS, in shrimp) routinely trigger mortality rates exceeding 50%, with economic losses in shrimp farming alone estimated at USD 1 billion annually [4,5,6,7]. Notably, multidrug-resistant *Vibrio* spp. and *Aeromonas* strains isolated from aquaculture have been detected in seafood and coastal environments, posing zoonotic risks. These diseases not only reduce production yields but also compel farmers to escalate antibiotic usage, creating a vicious cycle of antibiotic use, resistance development, and increased antimicrobial dependence. The overreliance on antibiotics to control these outbreaks has accelerated the global spread of multidrug-resistant bacteria, which now cause an estimated 1 million deaths per year [8,9]. Although many countries have imposed restrictions on antibiotic use, the development of safe and effective alternatives remains an urgent priority.

Antimicrobial peptides (AMPs), evolutionarily conserved components of innate immunity, have emerged as promising alternatives for breaking this cycle due to their multi-target mechanisms (e.g., membrane disruption, biofilm inhibition, and immunomodulation) and their low propensity to induce resistance [10,11]. Unlike traditional antibiotics, AMPs exhibit broad-spectrum activity against both Gram-positive and Gram-negative bacteria, including multidrug-resistant strains prevalent in aquaculture (e.g., *A. hydrophila*, *Aeromonas veronii*) [12,13,14,15]. However, challenges including high production costs, poor stability, and limited application scope have hindered their large-scale implementation. Recent studies demonstrate that AMPs can generate synergistic effects when combined with antibiotics, polysaccharides, herbal extracts, or AMPs [16,17,18,19]. Notably, such synergism not only strengthens antibacterial potency and broadens the antimicrobial spectrum but also mitigates the risk of resistance emergence.

This review aims to summarize recent advances in AMPs and their synergistic applications. It provides an overview of the molecular mechanisms, delivery systems, and application outcomes. Additionally, it examines current challenges and future directions to support the efficient and safe use of AMP-based synergistic strategies in aquaculture. The present review distinguishes itself from previously published works in several key aspects. Unlike earlier reviews that focus primarily on AMP classification or single-combination strategies, this review compares four distinct synergistic paradigms of four distinct synergistic paradigms within the specific context of aquaculture: AMP-antibiotic, AMP-polysaccharide, AMP-herbal extract, and AMP-AMP. We analyze the unique and shared mechanisms across these paradigms, and critically evaluate their translational potential from laboratory findings to practical aquaculture applications. Furthermore, we explicitly discuss the current limitations and future directions for each strategy, offering a roadmap for the rational design of next-generation AMP-based therapeutics to combat antimicrobial resistance in aquatic food systems.

## 2. Materials and Methods

This review employs a narrative synthesis approach to integrate evidence on the synergistic application of AMPs for controlling infectious diseases in aquaculture. The literature search was conducted up to 2026 across PubMed, Web of Science, Scopus, ScienceDirect, SpringerLink, and Google Scholar using keyword combinations including “aquaculture AMPs,” “AMPs and antibiotic combinations,” “AMPs and polysaccharide combinations,” “AMPs and herbal extract combinations,” and “AMPs combinations.” Of 673 initial records, 162 studies were included after screening titles, abstracts, and full texts. Studies were eligible if they described AMP structure/mechanisms, investigated AMPs in aquaculture species, focused on nano-formulated AMPs, or evaluated AMP combinations with antibiotics, polysaccharides, herbal extracts, or other AMPs. Conference abstracts, duplicate reports, non-peer-reviewed sources, and non-English publications were excluded. Owing to substantial heterogeneity among the included studies, a quantitative meta-analysis was not feasible; instead, a qualitative thematic analysis was performed, and eligible articles were organized into thematic sections by primary research focus.

## 3. AMPs

### 3.1. Classification and Structural Diversity of AMPs

**Figure 1:**
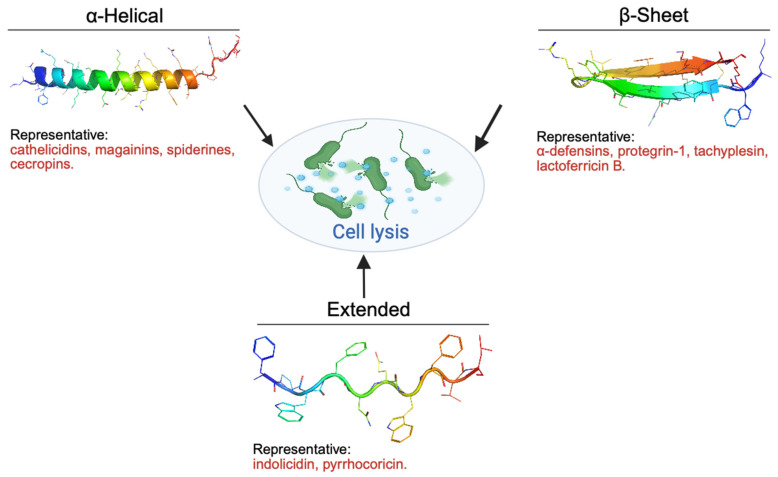
Classification and structural diversity of AMPs. AMPs are categorized based on their structural characteristics and sources. Structurally, AMPs are classified into three main types: α-helical peptides, β-sheet peptides stabilized by disulfide bridges, and extended peptides rich in specific amino acids.

AMPs are indispensable components of the host defense system and are widely distributed across the animal, plant, and microbial kingdoms [20]. Owing to their diverse structures and broad-spectrum antimicrobial activities, AMPs have emerged as promising candidates to combat multidrug-resistant bacteria, effectively circumventing the resistance issues associated with traditional antibiotics [21,22]. The structural heterogeneity of AMPs is reflected in their rich classification, primarily divided into three major categories based on structural characteristics (Figure 1): α-helical peptides (such as Cathelicidins [12], Magainins [23], Spiderines [24], Cecropins [25], and Piscidins from teleost [26]), which are rich in positively charged residues and form amphipathic α-helical structures upon membrane contact [27]; β-sheet peptides (such as α-Defensins [28], Protegrin-1 [29], Tachyplesin [30], and Lactoferricin B [31]), which are stabilized by disulfide bonds to form stable β-sheet structures; and extended peptides (such as Indolicidin [32] and Pyrrhocoricin [33]), which lack typical secondary structures but exhibit unique folding patterns upon interaction with membranes [34]. The charge distribution, hydrophobicity, and amphipathicity of AMPs significantly influence their antimicrobial efficacy.

### 3.2. Selective Antibacterial Mechanism Mediated by Membrane Composition Disparity

Most AMPs have both direct antibacterial activity and low toxicity toward host cells. This property arises primarily from the marked differences in membrane composition between host and bacterial cells [13,14,35,36]. Bacterial membranes are rich in negatively charged phospholipids, including phosphatidylserine (PS), phosphatidylglycerol (PG), and cardiolipin (CL) [14,37,38]. Additionally, surface lipopolysaccharides (LPS) on Gram-negative bacteria and lipoteichoic acids (LTA) in Gram-positive bacteria further enhance the negative charge of these membranes. In contrast, mammalian cell membranes are predominantly composed of electrically neutral phospholipids such as phosphatidylethanolamine (PE) and phosphatidylcholine (PC) [37,38]. Owing to their “cationic and amphipathic” structural features, positively charged AMPs bind to bacterial membranes through electrostatic adsorption and subsequently exert antibacterial effects through either membrane-dependent or membrane-independent pathways [15,39,40,41].

### 3.3. Dual Antibacterial Strategy via Membrane Disruption and Intracellular Targeting

**Figure 2:**
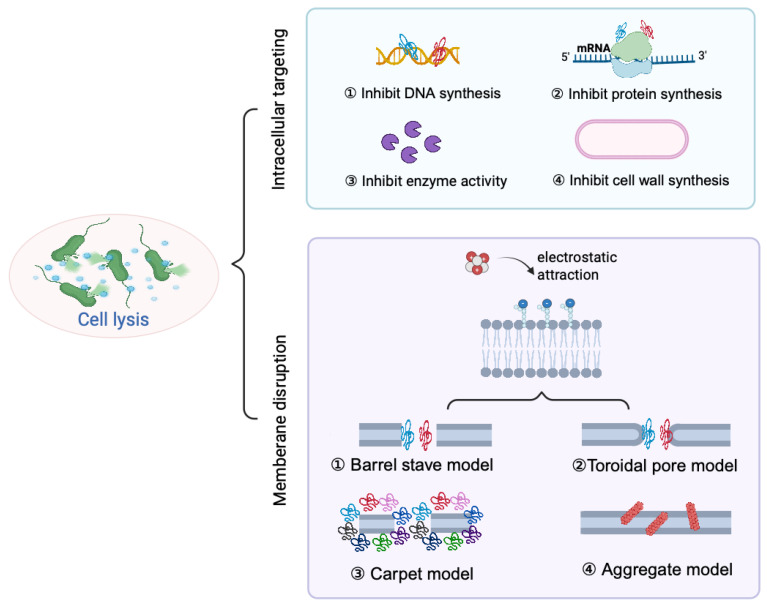
Schematic illustration of the two major antimicrobial mechanisms of AMPs. AMPs exert their bactericidal effects primarily through membrane disruption and intracellular targeting. The membrane disruption mechanisms include four classical models: barrel-stave, carpet, toroidal pore, and aggregate models, which lead to increased membrane permeability, pore formation, and eventual cell lysis. The intracellular targeting mechanisms involve inhibition of essential macromolecular processes, including DNA synthesis, protein synthesis, enzyme activity, and cell wall synthesis, ultimately disrupting bacterial growth and survival. These dual modes of action contribute to the broad-spectrum activity and low propensity for resistance development of AMPs.

AMPs exhibit potent antibacterial activity through two distinct yet complementary mechanisms: membrane-disruptive and intracellular-targeting pathways (Figure 2). This dual action strategy not only ensures broad-spectrum efficacy but also minimizes resistance development compared to traditional antibiotics that rely on single-target inhibition. The primary mode of action for many AMPs involves direct interaction with bacterial cell membranes. Positively charged AMPs are electrostatically attracted to negatively charged components such as LPS on Gram-negative bacteria or LTA on Gram-positive bacteria [39]. This interaction triggers membrane destabilization through four established biophysical models: barrel-stave pore formation (observed in α-helical Cecropin), toroidal pore formation (exemplified by Dermaseptin), carpet-like detergent disruption (characteristic of LL-37), and aggregate-induced non-specific channel formation (demonstrated by Protegrin) [42,43,44]. Each mechanism individually triggers cytoplasmic leakage and bacterial lysis. Beyond membrane disruption, AMPs employ intracellular strategies to impair bacterial viability through multiple pathways. For example, grass carp hepcidin directly binds to bacterial DNA and RNA to block replication and transcription processes [45]; zebrafish Pt5-1c inhibits *Staphylococcus aureus* by targeting the cell membrane with associated ribosomal perturbation [46]; the AMP NP-6 from pepper seeds inhibits the intracellular enzyme β-galactosidase activity of *Escherichia coli* in a dose-dependent manner [47]; and teixobactin inhibits cell wall synthesis through specific binding to lipid II and lipid III [48]. The synergistic combination of membrane disruption and intracellular inhibition contributes to broad-spectrum antibacterial efficacy while minimizing resistance development, because bacteria face a significantly higher metabolic burden when altering their membrane composition than when evolving single-target defenses.

### 3.4. Synergistic Strategies Overcome Drug Development Hurdles

Despite their unique membrane-lytic mechanisms and broad-spectrum activity, the practical translation of AMPs into aquaculture is hindered by several critical limitations. First, high production costs remain a major barrier [49]. Chemical synthesis of AMPs is expensive, particularly for long sequences, and recombinant expression systems often yield low quantities, making large-scale manufacturing economically unfeasible for the aquaculture industry. Second, poor in vivo stability severely limits practical applications. AMPs are rapidly degraded by proteolytic enzymes in aquatic environments and have short half-lives that diminish their effective concentrations before they reach target pathogens [49,50]. Third, safety concerns require careful evaluation. Although AMPs are generally considered safe due to their natural origin, excessive or prolonged exposure may overstimulate immune responses, induce inflammatory reactions, or cause cytotoxic effects in aquatic organisms. Furthermore, repeated application may result in residual peptide accumulation in animal tissues or environmental matrices, raising concerns about food safety and long-term ecological impacts [44,49,50]. AMPs may exert unintended effects on non-target beneficial microorganisms that play essential roles in nutrient cycling, organic matter degradation, and host-associated microbiota homeostasis. Continuous exposure to exogenous AMPs could disrupt the balance of commensal microbial communities in cultured animals and surrounding environments, thereby affecting host health and environmental stability. Such dysbiosis may indirectly impair immune competence and increase susceptibility to opportunistic infections [51,52].

Additionally, regulatory limitations further impede clinical translation. Currently, no standardized regulatory framework exists specifically for AMP-based products in aquaculture; most countries classify them under existing antimicrobial or feed additive guidelines, which were not designed for peptides. This creates uncertainty in approval pathways and residue tolerances. Delivery through feed or immersion poses practical hurdles, and the environmental fate of AMPs released into aquatic systems remains insufficiently understood. Although many AMPs are biodegradable, their persistence can vary depending on peptide structure, water chemistry, salinity, temperature, and microbial activity. Finally, farmer adoption challenges must be considered. High cost compared to conventional antibiotics, lack of familiarity with peptide-based therapies, and concerns about storage stability and application complexity may deter uptake, even if technical efficacy is proven. Importantly, the widespread and indiscriminate use of AMPs may also contribute to the evolution of AMP resistance. Increasing evidence suggests that certain microorganisms can develop adaptive resistance mechanisms, including membrane modification, efflux pump activation, proteolytic degradation, and biofilm formation [53,54,55]. Moreover, cross-resistance between AMPs and conventional antibiotics has been reported in some bacterial species, highlighting the potential risk of accelerating antimicrobial resistance evolution under selective pressure [53,54,55]. These limitations underscore why monotherapy with AMPs is often impractical in real farming settings.

Collectively, these drawbacks necessitate the development of AMP-based synergistic strategies. By combining AMPs with antibiotics, polysaccharides, herbal extracts, or other AMPs, synergistic effects enable enhanced antimicrobial efficacy at substantially reduced peptide concentrations. This dose reduction mitigates potential toxicity to cultured species, minimizes drug residues in aquatic products, and lowers overall treatment costs [16,17,18,19]. Moreover, combinatorial approaches can repurpose conventional antibiotics that were previously abandoned due to resistance or toxicity [16,17,18,19]. Therefore, synergistic strategies represent a promising approach to overcome the pharmacological and economic limitations of AMPs, accelerating their transition from laboratory research to cost-effective, sustainable therapies in aquaculture.

The quantitative evaluation of synergistic effects is typically assessed using the fractional inhibitory concentration index (FICI), calculated as follows: FICI = FIC_A_ + FIC_B_ = (MIC_A_ combination/MIC_A_ alone) + (MIC_B_ combination/MIC_B_ alone). Based on the FICI values, synergistic effects can be categorized into four classes: synergy (FICI ≤ 0.5), partial synergy (0.5 < FICI ≤ 1.0), indifference (1.0 < FICI ≤ 2.0), and antagonism (FICI > 2.0). All FICI values reported in this review are interpreted according to these established criteria.

## 4. The Synergistic Action Pathways of AMPs

### 4.1. Synergistic Antimicrobial Effects of AMPs and Antibiotics

#### 4.1.1. Potential of AMPs-Antibiotic Combination Strategies

Since the 1980s, aquaculture has rapidly expanded and emerged as one of the fastest-growing food production sectors worldwide [1]. However, this growth has been accompanied by an increased frequency of disease outbreaks, particularly bacterial diseases, which pose significant threats to the sustainable development of aquaculture. To combat these diseases, antibiotics have been extensively used in aquaculture for therapeutic and prophylactic purposes [56,57,58]. Commonly used antibiotics, such as tetracyclines, florfenicol, and trimethoprim or sulfamethoxazole, exhibit varying usage patterns across different countries and regions, and effective regulation is often lacking [59,60,61]. The indiscriminate or excessive use of antibiotics has brought about a series of serious problems. Firstly, it has promoted the emergence and spread of antibiotic resistance among common bacterial pathogens such as *Edwardsiella tarda* and *A. hydrophila*, making these pathogens more difficult to treat [62,63]. Secondly, this misuse has led to the presence of antibiotic residues in aquatic animal products, posing potential risks to human health [64,65]. Moreover, the widespread use of antibiotics disrupts the microbial balance in aquaculture environments, affecting water quality and ecological balance, thereby exacerbating the challenges of disease control [66]. Therefore, the prudent and responsible use of antibiotics, along with the exploration and development of alternatives, has become an urgent issue for the aquaculture industry.

Given the aforementioned challenges, the synergistic use of AMPs and antibiotics has emerged as a promising strategy to combat bacterial infections in aquaculture. Although most mechanistic insights into AMP-antibiotic synergy originate from mammalian models (Supplementary Table S1), these studies provide valuable mechanistic frameworks, such as membrane permeabilization, biofilm disruption, and efflux pump inhibition, that can guide research in aquatic species [16,67,68,69,70,71,72,73,74,75,76,77]. Importantly, recent studies in aquaculture species have begun to validate and extend these findings, as summarized in Table 1. Although the mammalian literature offers a strong foundation, direct evidence in fish, shrimp, and shellfish remains more limited, underscoring the need for species specific validation under real farming conditions.

#### 4.1.2. AMPs-Antibiotic Synergy in Aquaculture

In recent years, the synergy of AMPs-antibiotics has shown potential for aquaculture applications, with accumulating evidence summarized in Table 1. For example, LEAP-2, a liver-expressed AMP derived from teleost fish, exhibits strong synergistic activity with ampicillin against key aquaculture pathogens, including *Vibrio parahaemolyticus*, *Vibrio harveyi*, and *Bacillus subtilis* [78,79]. This combination reduces MICs by 4-fold, delays resistance development by up to 10 generations in vitro, and demonstrates feasibility for early-stage disease control [78,79]. In shrimp farming, when *V. harveyi* levels rise and shrimp vitality declines, the LEAP-2 and ampicillin combination can be applied to manage the outbreak. Similarly, the grass carp peptide C-I20, when paired with chloramphenicol, florfenicol, ampicillin, or enrofloxacin, further lowers MICs, suppresses resistance, and enhances pathogen clearance in high-density aquaculture systems [80]. Given the rapid pathogen transmission in such environments, C-I20-antibiotic combinations may stabilize ecosystems by improving bactericidal efficacy. Furthermore, hepcidin from starry flounder also demonstrates synergy with conventional antibiotics, further confirming the broad applicability of AMP-antibiotic synergy [81]. Additionally, PIS-A-1, derived from the fish AMP piscidin, acts synergistically with ampicillin [26], indicating that designing and optimizing AMPs is a viable strategy for discovering novel synergistic pairs. Collectively, these findings offer eco-friendly alternatives to traditional antibiotics, particularly for drug-resistant infections, although field validation remains essential.

Although these studies show promising synergistic effects, translating them into practical aquaculture applications requires addressing several critical challenges. Mechanistically, although the bactericidal effects of these combinations are known [26,78,79,80,81], the specific impacts on bacterial membrane permeability (including the order and mode of action), the precise target stages in protein synthesis, and the underlying signal-regulation pathways remain unclear. In terms of application scope, tailored strategies targeting pathogens specific to fish, shrimp, and shellfish have not yet been fully developed. Moreover, systematic research on long-term efficacy and safety (including impacts on the aquaculture ecosystem, farmed organism health, and human food safety) is lacking. Therefore, a multifaceted approach is needed to promote safe, efficient, and sustainable use of AMP-antibiotic combinations in aquaculture. On the mechanism front, advanced biotechnologies should be employed to precisely dissect changes in bacterial membrane permeability, identify target stages in protein synthesis, and elucidate signal regulation pathways, thereby strengthening the theoretical basis for optimizing combination therapies. Comprehensive long-term studies are also essential to assess ecosystem impacts, host health, and food safety. Ultimately, such research will guide the combined use of AMPs and antibiotics toward safe, efficient, and sustainable aquaculture practices.

In addition to these mechanistic and safety gaps, practical delivery remains a major hurdle. Oral administration via medicated feed is the most feasible route for most farmed fish and shrimp, but AMPs and antibiotics are susceptible to proteolytic degradation in the gut and may have poor intestinal absorption. Encapsulation in chitosan, alginate, or lipid-based microparticles can protect the active ingredients and enable sustained release. For example, chitosan encapsulated CATH-FLA has been shown to survive gastric conditions in trout [82,83]. Future research should evaluate AMP-antibiotic combinations in feed coatings or microencapsulated formulations under real farming conditions, including their stability during feed processing and storage.

**Table 1:** Synergistic antibacterial effects of AMPs and antibiotics in aquaculture.

AMP	Antibiotic	Target Pathogens	FICI	Observation(s)	References
C-I20 (Grass carp)	Chloramphenicol	*A. hydrophila*	ND	− C-I20 disrupts bacterial membrane structure and binds to DNA, enhancing antibiotic penetration and efficacy; Significant inhibition of bacterial growth when combined; delayed emergence of antibiotic resistance.	[80]
C-I20 (Grass carp)	Florfenicol	*A. hydrophila*	ND	− Significant inhibition of bacterial growth when combined; delayed emergence of antibiotic resistance.	[80]
C-I20 (Grass carp)	Ampicillin	*A. hydrophila*	ND	− Significant inhibition of bacterial growth when combined; reduced tissue bacterial loads and pathological changes; delayed emergence of antibiotic resistance; suppression of ampicillin-induced resistance.	[80]
C-I20 (Grass carp)	Enrofloxacin	*A. hydrophila*	ND	− Significant inhibition of bacterial growth when combined; delayed emergence of antibiotic resistance.	[80]
Hepcidin (Starry flounder)	Kanamycin	*V. alginolyticus* (ATCC17749)	0.5	− A synergistic effect was observed when Hepcidin was combined with kanamycin against *V. alginolyticus*.	[81]
Hepcidin (Starry flounder)	Kanamycin	*V. parahaemolyticus* (ATCC33847)	0.5	− The combination of Hepcidin and kanamycin resulted in a synergistic inhibition of *V. parahaemolyticus* growth.	[81]
Hepcidin (Starry flounder)	Kanamycin	*V. harveyi* (ATCC33866)	0.375	− Significant synergistic antibacterial activity was observed between Hepcidin and kanamycin against *V. harveyi*.	[81]
Hepcidin (Starry flounder)	Kanamycin	*V. anguillarum* (ATCC19264)	0.5	− Hepcidin combined with kanamycin showed a synergistic effect against *V. anguillarum*.	[81]
Hepcidin (Starry flounder)	Kanamycin	*E. tarda* (Et-CD)	0.375	− The combination of Hepcidin and kanamycin exhibited a synergistic inhibition of *E. tarda* growth.	[81]
Hepcidin (Starry flounder)	Kanamycin	*S. iniae* (ATCC29178)	0.375	− A synergistic antibacterial effect was observed when PsHepcidin was used in combination with kanamycin against *S. iniae*.	[81]
Hepcidin (Starry flounder)	Ampicillin	*V. vulnificus* (ATCC279562)	0.5	− The combination of PsHepcidin and ampicillin showed an additive effect against *V. vulnificus*.	[81]
Hepcidin (Starry flounder)	Ampicillin	*V. parahaemolyticus* (ATCC33847)	0.375	− The combination of PsHepcidin and ampicillin resulted in a synergistic inhibition of *V. parahaemolyticus* growth.	[81]
Hepcidin (Starry flounder)	Ampicillin	*V. harveyi* (ATCC33866)	0.5	− Significant synergistic antibacterial activity was observed between PsHepcidin and ampicillin against *V. harveyi*.	[81]
Hepcidin (Starry flounder)	Ampicillin	*V. anguillarum* (ATCC19264)	0.375	− PsHepcidin combined with ampicillin showed a synergistic effect against *V. anguillarum*.	[81]
Hepcidin (Starry flounder)	Ampicillin	*E. tarda* (Et-CD)	0.375	− The combination of PsHepcidin and ampicillin exhibited a synergistic inhibition of *E. tarda* growth.	[81]
Hepcidin (Starry flounder)	Ampicillin	*S. iniae* (ATCC29178)	0.375	− A synergistic antibacterial effect was observed when PsHepcidin was used in combination with ampicillin against *S. iniae*.	[81]
LEAP-2 (Olive flounder)	Ampicillin	*B. subtilis* (KCTC1021)	0.5	− The combination of LEAP-2 and ampicillin showed a synergistic effect against *B. subtilis*, significantly increasing antibacterial activity compared to individual treatments.	[78]
LEAP-2 (Olive flounder)	Ampicillin	*V. harveyi* (KCTC12724)	0.375	− A synergistic effect was observed when LEAP-2 was combined with ampicillin against *V. harveyi*, resulting in enhanced antibacterial efficacy.	[78]
LEAP-2 (Topmouth culter)	Ampicillin	*V. harveyi* (Not specified)	0.375	− Membrane permeabilization by LEAP-2 facilitates ampicillin uptake; The combination of topmouth culter LEAP-2 and ampicillin shows synergy against *V. harveyi*, with LEAP-2 boosting the bacteria’s sensitivity to ampicillin.	[79]
LEAP-2 (Topmouth culter)	Ampicillin	*V. parahaemolyticus*	0.5	− Membrane permeabilization by LEAP-2 facilitates ampicillin uptake; The combination of topmouth culter LEAP-2 and ampicillin shows synergy against *V. parahaemolyticus*, with LEAP-2 boosting the bacteria’s sensitivity to ampicillin.	[79]
PIS-1 (Hybrid striped seabass)	Ampicillin	*S. aureus* (ATCC 43300)	0.31	− The combination of PIS-1 and ampicillin improved antibacterial activity compared to individual treatments, but was less effective than the PIS-A-1 and ampicillin combination.	[26]
PIS-3 (Hybrid striped seabass)	Ampicillin	*S. aureus* (ATCC 43300)	0.31	− Similar to PIS-1, the combination of PIS-3 and ampicillin improved antibacterial activity but was less effective than the PIS-A-1 and ampicillin combination.	[26]
PIS-A-1 (Hybrid striped seabass)	Ampicillin	*S. aureus* (ATCC 43300)	0.19	− Enhanced membrane permeability, facilitating increased antibiotic uptake; The combination of PIS-A-1 and ampicillin showed strong bactericidal activity against methicillin-resistant *S. aureus*, utilizing PIS-A-1 that can be used as an antibiotic adjuvant to reverse methicillin-resistant *S. aureus* pathogens.	[26]

ND: Not detected; Synergy: FICI ≤ 0.5.

#### 4.1.3. The Synergistic Antimicrobial Mechanism of AMPs and Antibiotics

**Figure 3:**
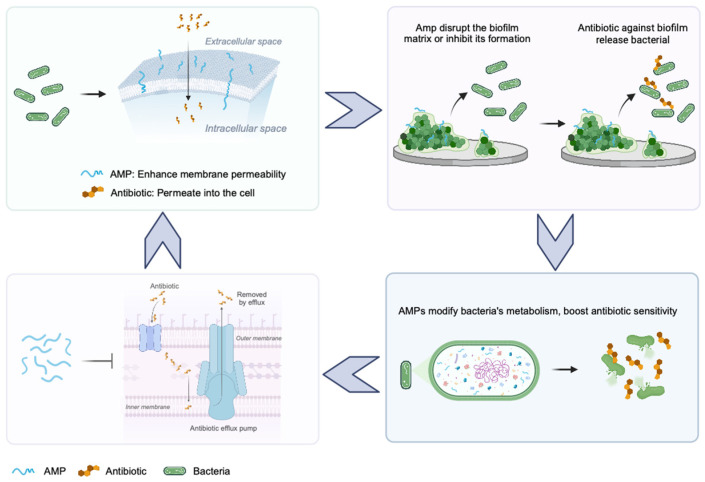
Proposed mechanisms of AMP-antibiotic synergy. AMPs and antibiotics cooperate to enhance antibacterial efficacy through multiple pathways. (1) AMPs increase bacterial membrane permeability, facilitating antibiotic entry into the intracellular space. (2) AMPs disrupt the biofilm matrix or inhibit its formation, releasing embedded bacteria and making them accessible to antibiotics. (3) AMPs modulate bacterial metabolism, thereby boosting bacterial sensitivity to antibiotics. These complementary actions collectively improve pathogen clearance and reduce the risk of resistance development.

Before focusing on aquaculture-specific evidence, it is important to note that the mechanistic understanding of AMP-antibiotic synergy, including membrane permeabilization, biofilm disruption, and metabolic modulation, has largely been established in mammalian models. These mechanisms are likely conserved across host species and thus provide a rational basis for their application in aquaculture. Antibiotics act through four primary mechanisms: inhibiting cell wall synthesis (e.g., penicillin, cephalosporins), disrupting membranes (e.g., polymyxin), interfering with protein synthesis (e.g., chloramphenicol, tetracyclines), and blocking nucleic acid transcription or replication (e.g., quinolones) [84,85,86,87,88,89,90]. AMPs enhance antibiotic efficacy by increasing bacterial membrane permeability, thereby enabling intracellular-acting antibiotics to penetrate cells more effectively (Figure 3) [91]. For example, LL-37 combined with colistin reduces the MICs of multidrug-resistant *E. coli* by permeabilizing membranes and bypassing efflux pumps [92]. Similarly, pleurocidin disrupts membranes and induces hydroxyl radicals, thereby amplifying antibiotic damage [93,94]. Moreover, PMAP-36/PRW4 combined with aminoglycosides further demonstrate that membrane permeabilization is critical for synergistic killing [16,95].

Concurrently, biofilms protect bacteria from antibiotics, whereas AMPs degrade or inhibit biofilm matrices to restore drug susceptibility (Figure 3). For instance, Cecropin A breaks down uropathogenic *E. coli* biofilms, enabling nalidixic acid to clear infections without resistance emergence [96]. Multiple AMPs (e.g., GVF27, Pt5-1c, LL-37), when combined with corresponding antibiotics, can effectively disrupt bacterial biofilms, restore or enhance bactericidal activity, and thereby combat multidrug-resistant bacteria [92,97,98]. In murine models, synthetic peptides combined with meropenem and erythromycin significantly reduce infections caused by ESKAPE pathogens (*Enterococcus faecium*, *S. aureus*, *Klebsiella pneumoniae*, *Pseudomonas aeruginosa*, and *Enterobacter* spp.), which are known for their biofilm-forming capacity [16,99]. Additionally, the synergy between AMPs and antibiotics extends to specific pathogens such as *Rhodococcus equi*, where Citropin 1.1 combined with rifampicin shows enhanced activity against its biofilms [70].

Additionally, AMPs enhance antibiotic potency through diverse mechanisms that alter bacterial physiology and metabolism (Figure 3). For example, BMAP-27B reverses carbapenem resistance in NDM-positive bacteria by inhibiting efflux pumps and chelating Zn^2+^, thereby blocking NDM protease activity [100]. Similarly, SLAY-P1 restores vancomycin efficacy in resistant *Enterococci* by suppressing the vanRS two-component system, a key resistance pathway [101]. In another strategy, Arenicin-1 induces oxidative stress through hydroxyl radical production and NADH depletion, amplifying the impact of co-administered antibiotics [102].

Furthermore, AMPs play a multifaceted role in combating antibiotic resistance (Figure 3). One key mechanism is the blockade of efflux pumps. For example, the synergy between the AMP D-11 and macrolides is attributed to increased membrane permeability and reduced efflux pump function, leading to enhanced intracellular antibiotic accumulation [103]. Similarly, combining the AMP derivative PMBN with efflux pump inhibitors significantly boosts the activity of azithromycin and renders *P. aeruginosa* susceptible to a broad range of antibiotics [104]. In addition to targeting efflux pumps, AMPs can directly inhibit enzymes involved in bacterial drug resistance. For instance, the AMPs Indopt 1-6 act synergistically with tobramycin and gentamicin by inhibiting aminoglycoside phosphotransferase and aminoglycoside acetyltransferase. These enzymes normally inactivate aminoglycosides through phosphorylation or acetylation, and their inhibition thereby restores antibiotic effectiveness [105,106].

### 4.2. Synergistic Antimicrobial Effects of AMPs and Polysaccharide

#### 4.2.1. Advantages of the Synergistic Action Between AMPs and Polysaccharides

Polysaccharides are abundant, biocompatible, and low-toxicity biopolymers derived from plants, microorganisms, and marine life. Some possess inherent antimicrobial and immunomodulatory activities, and can also serve as carriers to improve the delivery and stability of active ingredients. Due to their complementary functions, using polysaccharides as carriers for AMPs to achieve synergy has emerged as a promising strategy in aquaculture antibacterial research. This synergy offers several potential advantages for aquatic-disease control. Rational combinations of AMPs and polysaccharides may strengthen antimicrobial activity against key aquaculture pathogens, enabling more effective bacterial control. Certain polysaccharides could broaden the spectrum of action by facilitating AMP activity against a wider range of aquatic pathogens, including those less susceptible to AMPs alone. Polysaccharide carriers can also compensate for the limitations of individual AMP components, such as protecting AMPs from degradation in aquatic environments or the fish gastrointestinal tract, and reducing potential toxicity to cultured species. These potential benefits collectively support the development of sustainable antimicrobial alternatives in aquaculture.

#### 4.2.2. AMPs-Polysaccharide Synergy in Aquaculture

Polysaccharides such as chitosan and sodium alginate serve as effective carriers for AMPs, forming composite systems through physical encapsulation, chemical cross-linking, or electrostatic interactions. These systems enhance AMP stability and enable targeted release, thereby addressing key limitations of AMPs in aquaculture, including poor stability, susceptibility to enzymatic degradation, and low bioavailability. The synergistic antibacterial effects of various AMP-polysaccharide combinations in aquaculture are summarized in Table 2. For instance, chitosan, with its positively charged surface, forms nanoparticles with negatively charged AMPs such as Hepcidin and CATH-FLA through electrostatic adsorption [82,83]. These composites remain stable in the acidic gastric environment of grass carp and rainbow trout, but release AMPs upon entering the intestine as a result of the increased pH, thereby effectively inhibiting intestinal pathogens [82,83]. In contrast, sodium alginate forms hydrogels by cross-linking with calcium ions, encapsulating polymyxin B to create injectable wound dressings [107]. These dressings continuously release AMPs at fish wound sites, maintaining a moist environment that promotes cell proliferation and angiogenesis, thereby accelerating wound healing [107]. Carboxymethyl chitosan (CMCS) forms a structurally stable composite with the gcIFN-20H antimicrobial peptide through covalent binding, in which the carboxyl group of CMCS undergoes an amidation reaction with the amino group of the AMP [108]. This composite significantly enhances the resistance to infection of grass carp by regulating the expression of immune-related genes such as IL-1β and TNF-α [108]. Additionally, chitosan can load the Octominin antimicrobial peptide via physical encapsulation to prepare nanoparticles [109]. These particles form a protective layer at fish wound sites, continuously release AMPs, down-regulate the expression of inflammatory factors such as IL-6 and COX-2, and promote wound re-epithelialization [109]. These studies not only validate the antibacterial effects of antimicrobial peptide-polysaccharide composites but also reveal the molecular basis of their synergistic action through mechanisms such as regulating immune responses and promoting tissue repair.

In aquaculture, oral administration through medicated feed is the most practical and cost-effective route for delivering antimicrobial peptides, as it avoids the stress of injection and is suitable for mass treatment. However, the efficacy of oral AMPs is severely limited by gastric degradation and poor bioavailability. Polysaccharide based delivery systems have proven effective in overcoming these barriers. For example, chitosan microparticles encapsulating the rainbow trout peptide CATH-FLA remained intact under simulated gastric pH (2.0) and released the peptide at intestinal pH (8.0), leading to upregulation of immune genes in the head kidney and intestine [83]. Similarly, CMCS nanoparticles loaded with grass carp AMP gcIFN-20H showed sustained release over 20 h and resistance to trypsin degradation; oral administration of these nanoparticles significantly reduced mortality against *A. hydrophila* [108]. In addition, chitosan and alginate bioparticles carrying the spider-derived peptide JM72 exhibited pH-responsive release and high palatability when fed to ornamental fish, with no evidence of intestinal toxicity [110]. These examples highlight that polysaccharide-based oral delivery systems, ranging from simple feed mixing to nano-encapsulation, are viable strategies to translate AMPs into practical aquaculture applications.

Despite progress, research on AMP-polysaccharide synergy in aquaculture remains limited. Current studies focus on a limited range of combinations and lack exploration of diverse AMP types (e.g., linear, cyclic, and amphiphilic) and polysaccharides (e.g., chitosan derivatives, alginates, and hyaluronic acid). This restriction hampers the optimization of their combined performance. Moreover, inadequate evaluation of preparation processes (e.g., crosslinking degree, particle size) and stability (e.g., storage conditions, enzymatic degradation) undermines the consistency and effectiveness of AMP-polysaccharide complexes in large-scale production. Furthermore, although initial insights into the molecular mechanisms of AMP-polysaccharide synergism exist, a comprehensive understanding of key signaling pathways (e.g., MAPK and NF-κB) and regulatory networks (e.g., crosstalk between the microbiome and the immune system) is still lacking. To address these challenges, future studies should focus on four key areas: (1) expanding AMP-polysaccharide combinations to identify optimal pairs; (2) optimizing preparation processes for stable and scalable production; (3) investigating molecular mechanisms, including effects on fish immunity and microbiota, to enable precise antibacterial strategies; and (4) validating applications in real aquaculture settings to assess practical effects and economic feasibility. This will facilitate the transition from laboratory to production and promote sustainable aquaculture development.

**Table 2:** Synergistic antibacterial effects of AMPs and polysaccharides in aquaculture.

AMP	Polysaccharide	Formation Mode of Nanoparticles	Synergy Mechanism	Observation(s)	References
CATH-FLA (Rainbow trout)	Chitosan	Spray drying to form chitosan microparticles encapsulating CATH-FLA	− Chitosan microparticles protect CATH-FLA from gastric degradation, allowing sustained release in the intestinal portion. − Chitosan enhances the stability of CATH-FLA during storage and oral administration.	− Increased expression of immune-related genes in head kidney and intestinal tissues. − Enhanced antiprotease activity in the serum of treated fish. − Improved systemic immune response in rainbow trout.	[83]
Hepcidin (Grass carp)	Chitosan	NA	− Combined oral administration allows for sustained release of both hepcidin and chitosan in the gut, prolonging their effects. − Chitosan may enhance the stability of hepcidin in the gut environment, protecting it from degradation.	− Improved growth performance (WG, SGR, CF, HSI). − Enhanced survival rate against *F. columnare* infection. − Regulated iron metabolism and protected gill tissue from pathological damage. − Boosted innate immune response (elevated LZM, T-SOD activities). − Modulated gut microbiota (increased beneficial bacteria, decreased harmful bacteria).	[82]
IFN-20H (Grass carp)	Carboxymethyl chitosan (CMCS)	Ionic gelation method to form CMCS-20H nanoparticles	− CMCS-20H nanoparticles provide sustained release of gcIFN-20H, protecting it from enzymatic hydrolysis in the intestine. − CMCS enhances the stability of gcIFN-20H, allowing it to resist degradation in the gastrointestinal tract.	− Powerful antibacterial activity: CMCS-20H exhibits strong bactericidal effects against both Gram-negative and Gram-positive bacteria. − Increased survival rate and significantly reduced tissue bacterial loads after challenge. − Enhanced serum immune responses, including increased complement C3 content, lysozyme activity, and total superoxide dismutase activity. − Alleviated tissue lesions and inflammation: CMCS-20H reduces tissue damage and inflammatory cell infiltration, as shown by histological examination. − Improved mRNA expressions of immune-related genes. − Maintained intestinal microbiome homeostasis: CMCS-20H helps maintain a stable and healthy intestinal microbiome composition during bacterial infection.	[108,111]
JM72 (Spider)	Chitosan-N-arginine & Sodium alginate	Complex coacervation to form submicrometric polypeptide-bioparticles	− The bioparticle structure provides pH-responsive release of JM72, with low release in gastric conditions and effective release in intestinal conditions. − The bioparticle enhances the stability of JM72, protecting it from degradation and providing a controlled release mechanism.	− High palatability of the bioparticle formulation observed in fish, indicating good acceptance for oral administration. − Effective penetration of the bioparticle into intestinal tissues, as shown by fluorescence microscopy. − No cytotoxicity observed in intestinal tissues or blood cells.	[110]
Octominin (Octopus)	Chitosan	Ionic gelation method using carboxymethyl-chitosan (CMC) as an anionic cross-linker to form Octominin-CNPs	− Octominin-CNPs provide a controlled and sustained release of Octominin, enhancing its stability and bioavailability at the wound site. − Chitosan and CMC protect Octominin from degradation, ensuring its activity over an extended period.	− Reduced toxicity: Octominin-CNPs exhibit significantly lower toxicity compared to free Octominin in both in vitro and in vivo models. − Enhanced wound healing in vitro: Octominin-CNPs promote higher cellular proliferation and migration rates in HDF cells compared to free Octominin, as shown by the cell scratch assay. − Accelerated wound healing in vivo: Topical application of Octominin-CNPs on dermal wounds in zebrafish results in faster wound closure rates and higher wound healing percentages compared to controls. − Improved tissue regeneration: Histological analysis reveals reduced inflammation, enhanced re-epithelialization, and better tissue organization in Octominin-CNPs-treated zebrafish compared to controls. − Downregulated pro-inflammatory markers: Octominin-CNPs treatment leads to a significant reduction in the expression of pro-inflammatory cytokines and chemokines in zebrafish wound tissues, indicating reduced inflammation.	[109]

NA: Not applicable.

#### 4.2.3. The Synergistic Antimicrobial Mechanism of AMPs and Polysaccharides

**Figure 4:**
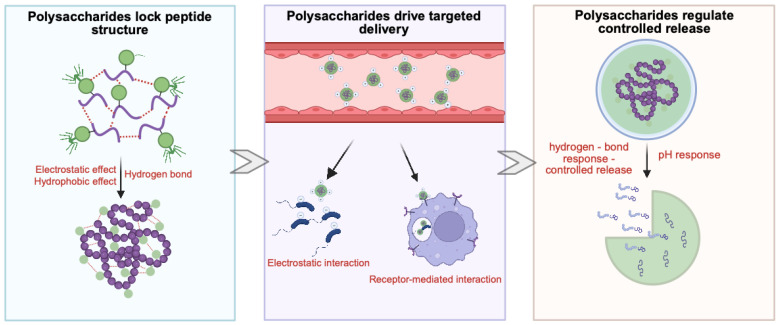
Proposed mechanisms of AMP-polysaccharide synergy. Polysaccharides enhance AMP efficacy through multiple mechanisms. (1) Polysaccharides lock AMP conformation via electrostatic interactions, hydrophobic effects, and hydrogen bonding, protecting AMPs from degradation. (2) Polysaccharide carriers enable precise delivery of AMPs to infection sites. (3) Polysaccharide matrices regulate AMP release in response to environmental triggers, including hydrogen-bond responsiveness and pH changes. (4) Electrostatic attraction and receptor-mediated interactions between polysaccharides and target cells further facilitate AMP accumulation and activity. These mechanisms collectively improve AMP stability, bioavailability, and therapeutic efficacy.

The mechanisms described below have been established primarily in mammalian, in vitro, or food models (Supplementary Table S2) [83,107,112,113,114,115,116,117,118,119,120,121]. They are presented here as a mechanistic framework that can guide the design of AMP-polysaccharide formulations for aquaculture. Physical and chemical interactions serve as the foundation for the stabilization of AMPs by polysaccharide carriers, and these mechanisms operate through interconnected mechanisms at multiple levels. Polysaccharides contain hydroxyl, carboxyl, and other active groups capable of forming hydrogen bonds, electrostatic interactions, hydrophobic interactions, or covalent linkages with AMPs, thereby stabilizing AMP conformation and preventing degradation (Figure 4). Specifically, anionic polysaccharides can generate electrostatic attraction with AMPs through their anionic groups (e.g., carboxyl groups), effectively enhancing complex stability [122,123]. For instance, the antimicrobial peptide Thanatin forms nanoparticles with hyaluronic acid and PLGA through electrostatic interactions, significantly enhancing its stability and prolonging its half-life [123]. Regarding hydrophobic interactions, the interaction between hydrophobic groups of AMPs and polysaccharides can enhance complex integrity [124]. For example, O-carboxymethyl chitosan (O-CMCS) and self-assembling peptides (SAPs) form a compact and stable complex through hydrophobic interactions and π-π stacking effects, enabling efficient loading and sustained release of the antimicrobial peptide Mel-d1 [124]. Hydrogen bonding is also of great importance, as the hydroxyl and amino groups in polysaccharides and AMPs can connect via hydrogen bonds, contributing to the formation and stability of nanodelivery systems and influencing AMP conformation and activity [116,125,126]. For example, the hydrogen bonding interaction between the antimicrobial peptide HX-12C and chitosan creates a uniform internal microstructure and reduces crystallinity, significantly affecting the physical, release, and functional properties of the chitosan-HX-12C complex, demonstrating its potential in pork preservation [116]. Additionally, AMPs can be covalently coupled with polysaccharides [127]. For instance, the antimicrobial peptide MSI-1 is covalently attached to a polyvinyl alcohol hydrogel modified with chitosan by coupling its primary amine group with a carboxyl group [127]. Further investigation of these interaction mechanisms will provide theoretical support and practical guidance for the development of more efficient and safe antimicrobial materials.

Polysaccharides (e.g., hyaluronic acid, chitosan, sodium alginate) address poor targeting efficiency and systemic toxicity by acting as biocompatible and biodegradable delivery carriers (Figure 4). Hyaluronic acid stands out in AMP delivery, owing to its high-affinity binding to CD44 receptor, which are expressed on macrophages and cancer cells. For instance, hyaluronic acid-LLKKK18 nanogels bind to CD44, boosting AMP stability and uptake to eradicate *Mycobacterium tuberculosis* more effectively [118]. In addition, chitosan modified hyaluronic acid-CM11 nanoparticles dually target drug-resistant bacteria and tumor cells via receptor-mediated endocytosis [112]. Beyond modifying other carriers, chitosan itself forms stable nanoparticles with negatively charged AMPs (e.g., NRC-07) via electrostatic interactions, protecting AMPs from enzymatic degradation and concentrating drug release at infection sites through charge-driven uptake [119]. Sodium alginate further expands the modalities of AMP delivery. For example, carboxymethyl chitosan and alginate composite hydrogels co-loaded with HHC36 AMP and silver nanoparticles combine membrane-penetrating targeting and synergistic effects for localized skin infection therapy [115]. Additionally, mannose functionalized AMP conjugates (e.g., Cip-CBT-Ada/CD-M) exploit multivalent lectin receptor interactions for macrophage-specific delivery and then self-assemble intracellularly into nanocomplexes, enhancing antibacterial activity while mitigating inflammation [128]. Collectively, these polysaccharide-based strategies enhance AMP bioavailability, therapeutic efficacy, and safety by improving infection-site enrichment and reducing off-target accumulation, paving the way for next-generation precision antimicrobials.

Polysaccharide carriers, owing to their unique degradation properties and environmental responsiveness, have become effective means to prolong AMP action time and achieve precise, sustained, and controlled release (Figure 4). Firstly, polysaccharide-based controlled release systems enhance AMP retention at target sites, thereby improving antimicrobial efficacy [114,117]. For instance, a hyaluronic acid and polyvinyl alcohol composite extends ε-poly-L-lysine release, enhancing long-term killing of *Propionibacterium acnes* [114]. Similarly, chitosan and LL-37 nanoparticles achieve 5-day sustained release of LL-37, significantly improving activity against methicillin-resistant *S. aureus* [117]. Secondly, pH-responsive controlled release is well exemplified by chitosan [112,116]. A chitosan and hyaluronic acid nanocarrier releases CM11 faster at acidic infection sites while limiting non-specific release in normal tissues, improving safety and efficacy [112]. In addition, chitosan-HX-12C composite films exhibit similar pH-dependent release behavior: under acidic conditions, protonated chitosan causes matrix swelling and increased electrostatic repulsion, enlarging pore size and promoting diffusion [129,130]; under neutral or alkaline conditions, deprotonation of chitosan amino groups reduces electrostatic repulsion, decreasing swelling and hindering content diffusion [131]; added salt ions shield electrostatic interactions between chitosan and HX-12C, suppressing swelling and HX-12C diffusion [129]. Furthermore, hydrogen bonding plays a key role in chitosan and CATH-FLA complexes. These complexes form stable microparticles in gastric acid (hydrogen bonds remain intact, preventing release), whereas the intestinal alkaline environment weakens hydrogen bonds, causing disintegration and precise release of CATH-FLA [83]. Future efforts can develop personalized antimicrobial delivery systems by tailoring polysaccharide carrier properties and AMP release parameters to match specific infection sites and pathogen types, thereby achieving more efficient, safer, and more highly targeted treatments.

### 4.3. Synergistic Antimicrobial Effects of AMPs and Herbal Extracts

#### 4.3.1. Synergistic Potential of Combining AMPs with Herbal Extracts

Herbal extracts are classified into primary and secondary metabolites based on their biosynthetic origin and functions [132]. Primary metabolites (e.g., chlorophyll, proteins) support basic cellular processes, whereas secondary metabolites, including terpenoids, phenolics, and nitrogen-containing compounds, serve as a diverse arsenal of bioactive molecules with inherent antimicrobial activities [132]. For instance, terpenoids such as thymol disrupt bacterial membrane integrity and metabolism [133,134]; artemisinin derivatives induce lethal oxidative stress [135]; phenolic compounds like curcumin and chlorogenic acid inhibit virulence pathways via quorum sensing interference and cause direct membrane damage [136,137,138,139,140]; and alkaloids such as berberine suppress nucleic acid synthesis [141,142]. This multifaceted mechanistic landscape establishes plant metabolites as ideal candidates for synergistic application with AMPs in aquaculture settings.

Recent studies have explored the combination of AMPs with herbal extracts for aquatic-disease control, demonstrating significant potential advantages in enhanced efficacy and improved safety (Table 3). For example, a system composed of curcumin and a degradable peptide polymer achieved a synergistic clearance rate exceeding 90% against aquaculture pathogens in experimental models, while the biodegradable nature of this combination avoided imposing selective pressure on environmental bacteria [19]. This synergy allowed for reduced individual component doses, thereby minimizing potential toxicity to cultured aquatic species. Furthermore, certain herbal polyphenolic components (e.g., curcumin) have been shown to improve the stability of AMPs in aqueous environments, addressing a key limitation of AMP application in aquaculture systems [143]. Collectively, these preliminary findings substantiate that the synergistic application of AMPs and herbal extracts holds promise for providing potent antibacterial activity against aquatic pathogens while optimizing safety, stability, and environmental compatibility.

#### 4.3.2. Synergistic Effect of AMPs and Herbal Extracts in Aquaculture

As shown in Table 3, AMPs and herbal extracts (e.g., curcumin) exhibit significant synergistic effects in aquaculture, including antibacterial and antiviral actions, as well as promising potential combinations. In terms of antibacterial action, the combination of poly(L-lysine)-r-poly(L-serine) and curcumin effectively inhibits pathogenic bacteria such as *V. fluvialis* and *S. agalactiae* [19]. The underlying mechanism is that curcumin reduces the negative charge on bacterial membranes, thereby facilitating AMP accumulation, accelerating membrane rupture, and causing cytoplasmic leakage, with FIC values both below 0.5 [19]. In a zebrafish infection model, this synergistic treatment increases survival to 66.7%, representing a more than 30% improvement over either agent alone [19]. For antiviral applications, the AMP I20 combined with curcumin directly interferes with viral replication by downregulating LMBV-MCP gene expression, while also boosting host immune responses via upregulation of IL-1β and IFN-γ [144]. Furthermore, antioxidant and anti-inflammatory properties of curcumin further enhance the antiviral effect [144]. Additionally, although not yet directly tested, curcumin and hepcidin present a promising synergistic partnership [145]. Curcumin minimizes oxidative stress, which can otherwise weaken host defense and increase vulnerability to pathogens. Hepcidin reduces bacterial iron availability, cutting off an essential nutrient for bacterial growth and survival. Together, these complementary mechanisms offer a promising strategy to improve disease resistance in cultured organisms [145].

Currently, research on the synergistic application of AMPs and herbal extracts in aquaculture is still in its infancy, and many synergistic mechanisms remain poorly understood. For instance, the molecular networks through which they regulate immune-related gene expression in aquatic animals have not been fully uncovered, impeding the development of precise antibacterial strategies. In the future, advanced molecular biology techniques such as transcriptomics and proteomics should be employed to unravel these complex molecular pathways, thereby laying a solid foundation for targeted antibacterial approaches. Regarding safety, existing assessments mainly focus on short-term in vitro and in vivo studies, and it remains unclear whether long-term use will lead to accumulation in aquatic animals or disrupt key physiological functions like the endocrine system. Long-term safety evaluation experiments should therefore be conducted to monitor accumulation in different tissues and perform comprehensive physiological and biochemical tests, with regulatory agencies establishing strict safety standards. Moreover, there is a lack of personalized protocols tailored to different aquaculture species, rearing conditions (e.g., water temperature and quality), and pathogens, making it difficult for farmers to apply and adjust these techniques. To address this, research institutions and the industry should collaborate on extensive field trials to build a database of optimal treatment parameters, develop intelligent decision support systems for personalized recommendations, and organize training programs to enhance understanding of farmers and enable them to make informed adjustments. Additionally, oral delivery of AMP-herbal combinations faces extra hurdles due to the poor water solubility and gut instability of many plant compounds. Encapsulation strategies that protect both partners can enhance bioavailability. For instance, curcumin has been successfully nano-encapsulated with AMPs in mammalian models, improving its stability and solubility [143]. Similar approaches could be adapted for aquaculture feeds. Future research should develop feed-compatible co-encapsulation systems (e.g., using lipid or polysaccharide carriers) that preserve the synergistic activity of AMP-herbal combinations under farm conditions.

**Table 3:** Synergistic antibacterial effects of AMPs and herbal extracts in aquaculture.

AMP	AMP Source	Herbal Extracts	Target Pathogens	FICI	Synergy Mechanism	References
Hepcidin	Grass carp	Grape seed proanthocyanidins (GSP); Berberine hydrochloride (BBR)	*A. hydrophila* (ATCC 7966)	ND	− GSP and BBR act as hepcidin agonists, significantly upregulating hepcidin and ferritin gene expression, enhancing iron regulation, and improving immune responses and resistance against bacterial infections in grass carp.	[145]
Poly(L-Lys)80-r-poly(L-Ser)20	Synthetic	Curcumin	*V. fluvialis* (CICC21612) *V. cholerae* (CICC23794) *S. agalactiae (CICC10465)*	<0.19 <0.5 <0.31	− Synergistic combo disrupts bacterial membranes via increased permeability, ATP release, and morphological changes; exhibits rapid bactericidal action, prevents resistance induction, and the biodegradable polymer avoids environmental pollution. In zebrafish models, it improves survival rates post-infection without significant toxicity.	[19]
I20H	Grass carp	Curcumin	Largemouth bass ranavirus		− The combination of P-I20H (expressed in Pichia pastoris) and curcumin significantly enhances the survival rate of largemouth bass infected with LMBV, clears the virus in tissues, upregulates immune gene expression (IL-1β, IFN-γ, Mx, IgM), boosts serum enzyme activities (TSOD, TAOC, C3), and mitigates tissue damage, indicating a synergistic effect in antiviral immunity and tissue protection.	[144]

Synergy: FICI ≤ 0.5.

#### 4.3.3. The Synergistic Antimicrobial Mechanism of AMPs and Herbal Extracts

**Figure 5:**
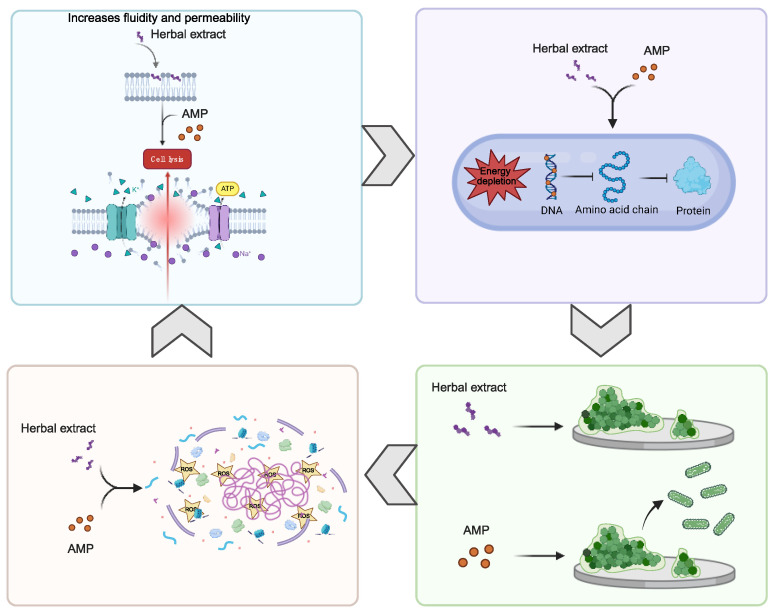
Proposed mechanisms of AMP-herbal extract synergy. Plant natural products and AMPs cooperate to exert bactericidal effects through multiple parallel pathways. Plant natural products (depicted in purple) increase bacterial membrane fluidity and permeability, facilitating AMP entry and promoting cell lysis. Additionally, plant natural products act jointly with AMPs to deplete intracellular ATP and inhibit bacterial DNA and protein synthesis (as indicated by the disruption of DNA and amino acid chains). They also inhibit biofilm formation and disrupt existing biofilms, while inducing or potentiating ROS production to cause oxidative damage.

The mechanisms summarized here have been largely established in mammalian and in vitro models (Supplementary Table S3) [146,147,148,149,150,151,152,153,154]. They provide testable hypotheses for aquaculture research, but direct validation in fish and shellfish remains limited. The synergistic antibacterial action of AMPs and herbal extracts follows a sequential process, starting from the disruption of physical barriers and progressing to the interference with internal biochemical systems. This cooperative mechanism is not a random process but rather an orchestrated, multi-step, and multi-target attack network (Figure 5).

The synergistic antibacterial action of AMPs and herbal extracts starts with physical disruption of the bacterial cell envelope (Figure 5). Specifically, hydrophobic phytochemicals (e.g., thymol, eugenol) integrate into the cytoplasmic membrane, increasing its fluidity and permeability. This initial disruption enables cationic AMPs (e.g., KRR-N1-5W6L, CF-14) to insert more rapidly and deeply into the membrane, forming stable transmembrane pores [147,149]. For methicillin-resistant *S. aureus*, thymol first disrupts the cell wall, causing leakage of alkaline phosphatase. Subsequent addition of KRR-N1-5W6L exacerbates membrane damage, leading to efflux of intracellular components such as K^+^ and ATP. Ultimately, scanning electron microscopy reveals severe cell adhesion, deformation, and rupture. This sequential “softening then piercing” collaboration forms the physical basis for the synergistic enhancement [147].

After disrupting the physical barriers, the synergistic assault rapidly shifts to the internal energy system and core metabolism of bacteria (Figure 5) [147,149]. Loss of plasma membrane integrity directly disrupts the proton-motive force, a vital factor for bacterial survival. For instance, thymol and the peptide KRR-N1-5W6L act together to dissipate both the transmembrane electric potential (Δψ) and the proton gradient (ΔpH) in methicillin-resistant *S. aureus*, effectively depleting bacterial energy metabolism and causing cell death via energy exhaustion [147]. Simultaneously, the increased membrane permeability allows other agents to enter the cell and interfere with metabolic processes [147,149]. When combined with KRR-N1-5W6L, thymol enhances the binding capacity between peptide and DNA, which likely disrupts key bacterial processes such as gene expression and DNA replication, thereby contributing to cell death [147]. Similarly, eugenol has been shown to increase the ability of the antimicrobial peptide CF-14 to bind to bacterial DNA, leading to more effective inhibition of DNA replication and transcription [149].

In addition to disrupting physical barriers and metabolism, the synergistic strategy also targets bacterial biofilms (Figure 5). Phytochemicals act as quorum sensing inhibitors, preventing bacterial aggregation and biofilm formation. For instance, curcumin effectively inhibits the quorum sensing system of *S. aureus*. When combined with a degradable peptide polymer, it not only reduces biofilm formation but also uses polymer degradation to physically disrupt existing biofilm matrices, thereby exposing embedded bacteria [19]. Furthermore, the synergy between curcumin and azithromycin has been shown to significantly degrade methicillin-resistant *S. aureus* biofilms [148]. This combined strategy of inhibiting biofilm formation and promoting physical biofilm clearance effectively dismantles the collective defense mechanism of bacterial communities.

At the molecular level, the synergistic action also disrupts bacterial intracellular redox homeostasis, exerting lethal chemical pressure (Figure 5) [147]. Some plant polyphenols (e.g., curcumin, quercetin) exhibit pro-oxidant activity within bacterial cells. However, thymol itself does not directly induce reactive oxygen species (ROS). Instead, it significantly potentiates the ability of the AMP KRR-N1-5W6L to generate ROS in methicillin-resistant *S. aureus*, leading to oxidative damage to bacterial proteins, lipids, and DNA [147]. Similarly, synergistic treatment with curcumin and azithromycin markedly increases intracellular ROS levels, confirming a ROS-mediated antibacterial mechanism [148]. This oxidative assault, combined with concurrent membrane damage and metabolic interference, delivers a multifaceted antibacterial effect to bacterial cells.

Finally, advanced delivery systems enable “performance synergy” by optimizing all the aforementioned biochemical mechanisms. Although this nanodelivery-based strategy does not introduce a new bactericidal mechanism, it ensures the full realization of multi-tiered synergy by enhancing bioavailability and enabling targeted delivery to infection sites. This improves physical membrane damage, metabolic interference, biofilm disruption, and oxidative assault. To overcome poor solubility and instability of components such as curcumin, AMPs or their derivatives can serve as building blocks for functional delivery vehicles [143,152]. For instance, the cell-penetrating peptide pVEC forms stable nanocomplexes with curcumin via non-covalent interactions, increasing aqueous solubility of curcumin by an order of magnitude and significantly improving its kinetic stability, thereby amplifying antibacterial activity [143]. Similarly, co-encapsulation of curcumin and the AMP LL-37 within chitosan nanoparticles enhances delivery efficiency of both agents and demonstrates improved antibacterial and gut microbiota-modulating effects in vitro and in vivo [152].

### 4.4. Synergistic Antimicrobial Effects of AMPs and AMPs

#### 4.4.1. Synergistic Potential of Combining AMPs with AMPs

The combined use of two or more AMPs represents a promising strategy for combating bacterial diseases. Such combinatorial strategies can enhance antimicrobial potency against aquatic pathogens, broaden the spectrum of activity, and reduce the risk of resistance emergence. By leveraging complementary mechanisms (e.g., membrane disruption combined with intracellular targeting), AMP-AMP synergy can achieve effective killing at lower individual peptide concentrations, thereby improving safety and cost-effectiveness for farmed aquatic species. Collectively, these advantages make AMP-AMP synergy a promising approach for sustainable disease management in aquaculture.

#### 4.4.2. AMP-AMP Synergy in Aquaculture

Although mechanistic studies of AMP-AMP synergy have been primarily conducted in mammalian and other model systems, emerging evidence suggests that similar synergistic interactions also occur in aquaculture species (Table 4). In Pacific oysters (*Crassostrea gigas*), the combination of the defensin Cg-Def and the proline-rich peptide Cg-Prp significantly reduces the MIC against *E. coli* and *Micrococcus lysodeikticus*, resulting in enhanced antimicrobial activity [155]. Further studies in oysters reveal that the coordinated action of distinct AMP families, such as cysteine-stabilized αβ (CSαβ)-containing defensins, Cg-BPI (a bactericidal/permeability-increasing protein), and Cg-Prp, expands the antimicrobial spectrum and enhances potency [156]. Specifically, Cg-BPI compromises the outer membrane of Gram-negative bacteria by inducing membrane lysis, while Cg-Defs target Gram-positive bacteria by inhibiting peptidoglycan synthesis; their combined use thus covers a broader range of pathogens [156]. Although Cg-Prp displays limited antimicrobial activity individually, it enhances the efficacy of both Cg-BPI and Cg-Defs, potentially by facilitating membrane penetration and interfering with bacterial metabolic processes [156]. Moreover, upon *V. splendidus* infection, oysters enhance their survival by modulating AMP expression and exploiting tissue-specific distribution (e.g., high Cg-Def expression in gills) and co-localization within hemocytes, thereby establishing a spatiotemporal functional network [157]. In addition to oysters, the combination of winter flounder-derived peptides WF1a and WF2 exerts broad-spectrum inhibition by disrupting the hydrogen bond network and altering the penetration depth within the bacterial membrane, leading to disorder of lipid acyl chains [158].

Building on these in vitro synergistic mechanisms, AMP combinations hold significant promise for enhancing therapeutic efficacy and mitigating antibiotic resistance in aquaculture. However, research on their synergistic effects in aquatic species remains scarce, which limits our understanding of how to optimally apply them in practice. To translate these findings into practical use, more fundamental multidisciplinary studies are urgently needed, particularly using robust animal models to elucidate the complex modes of action of AMP combinations, including their interactions with each other and with the host immune system. Strategically, such combinations can broaden the antimicrobial spectrum and potentiate activity at lower individual concentrations, thereby minimizing the selective pressure that drives resistance. Nevertheless, developing these combination therapies introduces considerable complexity, requiring meticulous analysis of formulation, dosage, administration routes, and pharmacokinetics for each peptide component to ensure efficacy and safety in aquatic species. Ultimately, the transition from laboratory research to commercial aquaculture treatments will depend on overcoming these pharmacological challenges through targeted in vivo studies. Oral delivery of two AMPs together presents unique challenges, such as maintaining their synergistic ratio during feed processing and gastrointestinal transit. Layer-by-layer coating or co-encapsulation in multi-compartment nanoparticles could address this issue. Although no aquaculture study has yet tested such formulations, the successful oral delivery of single AMPs via chitosan or alginate carriers provides a foundation [83,108]. Future research should explore these technologies for AMP-AMP pairs, with attention to cost-effective production of both peptides for commercial feed applications.

#### 4.4.3. The Synergistic Antimicrobial Mechanism of AMPs and AMPs

Given that the mechanistic basis of AMP-AMP synergy remains poorly characterized in aquaculture species, evidence from mammalian, amphibian, and other model systems can provide valuable insights into the fundamental principles governing these interactions (Supplementary Table S4) [159,160,161,162,163,164,165,166,167,168,169,170,171,172,173,174,175,176,177,178,179,180,181,182,183,184,185,186]. These mechanisms, which include structural complementarity, multi-target attack, and host immunomodulation, are likely applicable to aquatic species and can guide future research in aquaculture.

**Figure 6:**
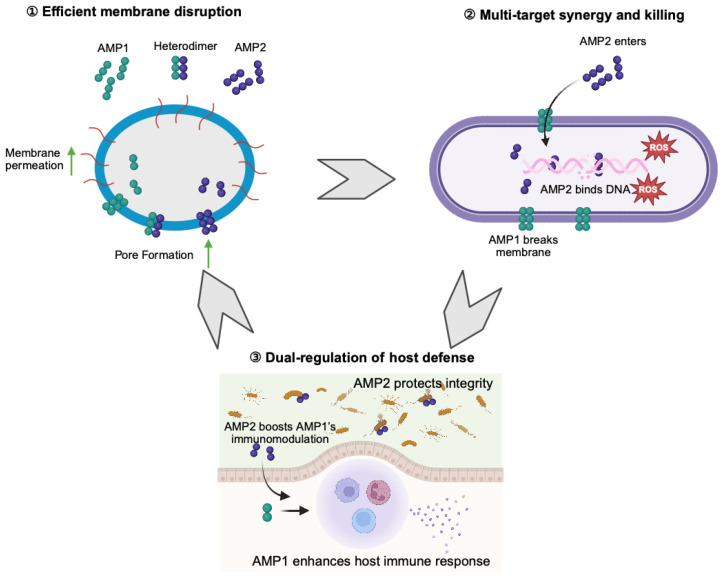
Proposed mechanisms of AMP-AMP synergy. AMPs cooperate through multiple mechanisms to achieve enhanced antibacterial activity. (1) AMP1 and AMP2 form a heterodimer that promotes membrane permeation and pore formation, leading to increased membrane permeability. (2) AMP1 disrupts the bacterial membrane, allowing for AMP2 to enter the cell. Once inside, AMP2 triggers ROS production, causing oxidative damage and cell death. (3) AMP1 enhances host immune responses, while AMP2 boosts the immunomodulatory activity of AMP1 and protects epithelial integrity. These coordinated actions collectively achieve potent bacterial killing and host protection.

The remarkable structural diversity of AMPs, including α-helical, β-sheet, and extended conformations, provides a molecular basis for synergistic action through structural complementarity (Figure 6). For instance, Magainin 2 (α-helix) and PGLa (random coil in solution) are both isolated from frog skin [23,187,188,189]. Notably, PGLa undergoes a conformational transition from random coil to α-helix upon interacting with bacterial membranes, reaching approximately 80% α-helix content. Individually, Magainin 2 and PGLa can form pores but have limited antibacterial effects. However, when combined at an equimolar ratio, they display a nearly ten-fold increase in antimicrobial potency [187,190,191]. This enhanced activity is attributed to the formation of a stable heterodimeric complex through non-covalent interactions, which promotes more efficient pore formation and membrane destabilization, ultimately leading to rapid bacterial cell lysis [187,192,193,194,195]. Similar synergistic effects have been reported for other structurally distinct AMP combinations. For example, the α-helical Magainin 2 mainly forms pores to disrupt the bacterial cell membrane, while the β-sheet Tachyplesin I readily penetrates bacterial membranes [23,187]. Their combined use shows strong synergistic activity against both Gram-negative and Gram-positive bacteria, mainly through a synergistic enhancement of membrane-disrupting effects related to the permeability of acidic phospholipid membranes [189,196]. Moreover, charge distribution also plays a role in the synergistic mechanisms of AMPs. Although SynSaf-P8 alone lacks membrane-disruptive activity, it significantly enhances the antimicrobial efficacy of the strongly membranolytic peptide SynSaf-P96, enabling effective bacterial killing at substantially lower concentrations [183]. The synergistic mechanism may involve SynSaf-P8 altering the local charge distribution of the bacterial membrane, thereby facilitating the more efficient pore formation and membrane disruption by SynSaf-P96 [183].

Beyond membrane disruption, many AMPs can penetrate bacterial cells and interfere with intracellular processes, including DNA replication, RNA transcription, and protein synthesis (Figure 6). Combining two or more AMPs allows simultaneous attack on multiple targets, enhancing overall efficacy. For example, AW1 from *A. wuyiensis* has broad-spectrum activity, while AW2 lacks direct antimicrobial activity but carries a strong positive charge and has a random coil structure [161]. When combined, AW2 markedly enhances the antibacterial activity of AW1, suppresses resistance development, and inhibits biofilm formation [161]. Mechanistic studies suggest that AW1 and AW2 cooperatively disrupt bacterial membranes, creating entry routes that facilitate intracellular access of AW2. AW2 binds genomic DNA and promotes excessive ROS production. When ROS accumulation exceeds the bacterial antioxidant defense capacity, extensive oxidative damage ultimately results in cell death [161]. Transcriptomic analyses further support this mechanism, revealing substantially more differentially expressed genes in the combination treatment than in either single-peptide treatment. These genes are enriched in pathways associated with ribosomal function, ABC transporters, and oxidative respiration, indicating widespread disruption of multiple intracellular processes [161]. These changes in gene expression are consistent with the direct impact of AW1 and AW2 on multiple intracellular bacterial processes, which collectively contribute to the enhanced antimicrobial effect.

In addition to directly targeting bacterial cells, certain AMP combinations exert synergistic effects through modulation of host immune responses (Figure 6). Human defensin 5 (HD-5) and defensin 6 (HD-6) exemplify this mechanism [169]. HD-5 has potent antimicrobial activity; it kills bacteria and stimulates intestinal epithelial cells to secrete interleukin-8 (IL-8), which recruits immune cells to infection sites [197,198,199]. However, HD-5 may also reduce transepithelial electrical resistance (TER), compromising epithelial barrier function [169]. HD-6 has limited direct antimicrobial activity but synergizes with HD-5 by enhancing HD-5-induced IL-8 secretion, thereby amplifying the immune response [169,199,200]. Moreover, HD-6 counteracts the HD-5-induced TER reduction, protecting epithelial barrier integrity and preventing bacterial invasion [169]. Thus, this synergy simultaneously boosts immune defense and preserves barrier function, collectively reinforcing host protection against bacterial infection.

### 4.5. Comparative Analysis of Synergistic Paradigms and Structural Drivers of Synergy

Four major categories of synergistic partners have been investigated in combination with AMPs, including antibiotics, polysaccharides, herbal extracts, and other AMPs. Each synergistic strategy exhibits distinct advantages and is therefore suited to different application scenarios in aquaculture. AMP-antibiotic combinations generally exhibit the strongest and fastest bactericidal activity, making them suitable for acute disease outbreaks caused by multidrug-resistant pathogens, but they carry a residual risk of resistance selection [78,79]. AMP-polysaccharide combinations offer superior stability and sustained release, ideal for prophylactic or long-term dietary supplementation, albeit with lower overall antimicrobial potency [117]. AMP-herbal extract combinations achieve multi-target attacks, including oxidative stress induction and quorum sensing interference, which is particularly valuable for antiviral applications and environmentally sensitive settings [144,145]. AMP-AMP combinations often exhibit strong synergistic antimicrobial activity without the need for additional chemical agents, but their adoption is constrained by production costs and limited validation in farmed species beyond oysters and flounder [155,156]. Overall, no single synergistic strategy is universally optimal, and the selection of an appropriate AMP-based combination should depend on the target pathogen, farming conditions, administration route, and regulatory considerations.

Several structural features of AMPs correlate with strong synergy across these paradigms. High net positive charge (+6 to +9) facilitates electrostatic interactions with bacterial membranes and also promotes binding to anionic polysaccharides or polyphenols [27,201]. Amphipathic α-helical or β-sheet conformations enable efficient membrane insertion and pore formation, which are critical for the synergistic mechanisms observed in AMP-antibiotic and AMP-AMP combinations, such as grass carp C-I20 [80]. High hydrophobicity promotes stronger membrane disruption and better synergy with hydrophobic herbal compounds such as curcumin and thymol [147,149]. In AMP-polysaccharide complexes, abundant arginine or lysine residues enhance electrostatic complexation formation with carboxylated polysaccharides, thereby improving protection and sustained release [82,83]. Collectively, these structure-activity relationships provide important theoretical guidance for the design and selection of AMPs tailored to specific synergistic applications in aquaculture.

## 5. Conclusions and Future Perspectives

AMPs, as naturally occurring antimicrobial molecules, have emerged as promising alternatives to conventional antibiotics for addressing the global antimicrobial resistance crisis. Their unique mechanisms of action, broad-spectrum antimicrobial activity, and relatively low propensity for inducing resistance make them attractive candidates for disease control in aquaculture. This review summarizes the classification and structural diversity of AMPs, as well as their dual antimicrobial strategies involving membrane disruption and intracellular targeting. Furthermore, the review highlights the synergistic antimicrobial effects achieved when AMPs are combined with antibiotics, polysaccharides, herbal extracts, or other AMPs. Accumulating evidence has demonstrated that AMP-antibiotic combinations can produce significant synergistic effects through multiple mechanisms, including enhanced membrane permeability, biofilm disruption, and modulation of bacterial metabolism, thereby improving antimicrobial efficacy while reducing the likelihood of resistance development. Similarly, the incorporation of AMPs into polysaccharide-based delivery systems can enhance peptide stability and bioavailability through physical protection, targeted delivery, and immunomodulatory effects. Synergistic interactions between AMPs and herbal extracts further strengthen antimicrobial activity via multi-target antibacterial mechanisms, oxidative stress induction, and biofilm degradation. In addition, combinations of different AMPs may achieve enhanced efficacy through structural complementarity, simultaneous multi-target actions, and modulation of host immune responses. Collectively, these synergistic strategies provide promising alternatives for controlling antibiotic-resistant pathogens in aquaculture and offer innovative approaches to reduce the excessive reliance on conventional antibiotics.

Despite the rapid expansion of research on AMP synergistic strategies, several important challenges and knowledge gaps remain. Much existing work has been conducted in vitro or in mammalian models, with relatively limited validation in aquatic species under practical farming conditions. Moreover, although the molecular mechanisms of individual AMP-partner combinations have been partially elucidated, a systematic understanding of how multiple synergistic pathways, including membrane permeabilization, biofilm disruption, metabolic modulation, and immune regulation, integrate simultaneously within a single combination is still lacking. Long-term ecological safety evaluations, such as impacts on non-target organisms, gut microbiome homeostasis, and the development of resistance under chronic low-dose exposure, remain largely unexplored. Furthermore, the application of omics technologies and systems biology approaches for the rational design of AMP synergistic combinations tailored to specific aquaculture pathogens and host species is still at an early stage.

Addressing these limitations will require a transition from empirical screening approaches toward mechanism-driven rational design strategies. Future studies should integrate high-throughput omics technologies, systems biology, artificial intelligence-assisted predictive approaches, and advanced biomaterial engineering to optimize synergistic AMP combinations for specific pathogens, host species, and aquaculture environments. Equally important is the establishment of standardized evaluation systems that assess not only short-term antimicrobial efficacy but also long-term ecological safety, pharmacokinetics, environmental fate, and resistance evolution under realistic farming conditions. Moreover, cost-effective production of AMPs and scalable formulation processes must be prioritized to ensure economic viability for the aquaculture industry. Beyond these downstream considerations, upstream efforts in AMP discovery and optimization are equally critical. Future integration of marine-derived AMP databases and peptide engineering approaches (e.g., sequence optimization, cyclization, and hybrid design) will further accelerate the rational development of synergistic combinations for aquaculture. Ultimately, promoting multidisciplinary integration of microbiology, immunology, materials science, and aquaculture engineering will be key to developing sustainable, safe, and efficient AMP-based synergistic therapies. This will help combat antimicrobial resistance while securing the health and productivity of aquatic food systems.

## Figures and Tables

**Table 4:** Synergistic antibacterial effects of AMPs and AMPs in aquaculture.

AMP1	AMP2	AMPs Source	Target Pathogens	FICI	References
BPI	lgPrp	Oyster	*E. coli* (SBS 363)	0.5	[157]
BPI	Defh1	Oyster	*M. lysodeikticus* (CIP5345) *E. coli* (SBS 363)	0.45 0.26	[157]
BPI	Defh2	Oyster	*M. lysodeikticus* (CIP5345) *E. coli* (SBS 363)	0.23 0.27	[157]
BPI	Defm	Oyster	*M. lysodeikticus* (CIP5345) *E. coli* (SBS 363)	0.23 0.31	[157]
Defh1	Defh2	Oyster	*M. lysodeikticus* (CIP5345)	0.5	[157]
Defh1	lgPrp	Oyster	*M. lysodeikticus* (CIP5345) *E. coli* (SBS 363)	0.35 0.5	[157]
Defh2	Defm	Oyster	*M. lysodeikticus* (CIP5345) *E. coli* (SBS 363) *V. splendidus* (CIP 107715)	0.37 0.5 0.5	[157]
Defh2	lgPrp	Oyster	*M. lysodeikticus* (CIP5345) *E. coli* (SBS 363)	0.3 0.28	[157]
Defm	lgPrp	Oyster	*M. lysodeikticus* (CIP5345)	0.45	[157]
lgPrp	lgPrp P/Q	Oyster	*M. lysodeikticus* (CIP5345)	0.5	[157]
Prp	Def	Oyster	*E. coli* (SBS 363)	0.29	[155]
Prp_22–36_	Def	Oyster	*M. lysodeikticus* (CIP5345)	0.32	[155]
Prp_26–36_	Def	Oyster	*M. lysodeikticus* (CIP5345)	0.33	[155]
WF1	WF1a	Winter flounder	*A. baumannii* (AYE)	0.5	[158]
WF1	WF1a-1	Winter flounder	*S. aureus* (NCTC 13616) *A. baumannii* (AYE)	0.5 0.5	[158]
WF1	WF2	Winter flounder	*S. aureus* (NCTC 13616) *K. pneumoniae* (NCTC 13368) *A. baumannii* (AYE)	0.19 0.31 0.5	[158]
WF1	WF3	Winter flounder	*S. aureus* (NCTC 13616) *K. pneumoniae* (NCTC 13368) *A. baumannii* (AYE)	0.19 0.5 0.25	[158]
WF1a	WF1a-1	Winter flounder	*S. aureus* (NCTC 13616) *K. pneumoniae* (NCTC 13368) *A. baumannii* (AYE)	0.38 0.25 0.25	[158]
WF1a	WF2	Winter flounder	*S. aureus* (NCTC 13616) *K. pneumoniae* (NCTC 13368) *A. baumannii* (AYE)	0.19 0.13 0.25	[158]
WF1a	WF3	Winter flounder	*S. aureus* (NCTC 13616) *A. baumannii* (AYE)	0.19 0.5	[158]
WF1a	WF4	Winter flounder	*A. baumannii* (AYE)	0.5	[158]
WF1a-1	WF3	Winter flounder	*K. pneumoniae* (NCTC 13368) *A. baumannii* (AYE)	0.5 0.19	[158]
WF2	WF3	Winter flounder	*K. pneumoniae* (NCTC 13368) *A. baumannii* (AYE)	0.25 0.25	[158]
WF2	WF4	Winter flounder	*S. aureus* (NCTC 13616) *A. baumannii* (AYE)	0.25 0.5	[158]
WF3	WF4	Winter flounder	*K. pneumoniae* (NCTC 13368) *A. baumannii* (AYE)	0.25 0.19	[158]

Synergy: FICI ≤ 0.5.

## Data Availability

No new data were created or analyzed in this study. Data sharing is not applicable to this article.

## Supplementary Materials

The following supporting information can be downloaded at https://www.mdpi.com/article/10.3390/ani16121774/s1. Table S1: Synergistic antibacterial effects of AMPs and antibiotics in mammals; Table S2: Synergistic antibacterial effects of AMPs and polysaccharides in mammals; Table S3: Synergistic antibacterial effects of AMPs and herbal extracts in mammals; Table S4: Synergistic antibacterial effects of AMPs and AMPs in mammals.
