# Supplementary material for: Potential of Antimicrobial Peptide Synergies for Combating Infectious Diseases in Aquaculture: A Review
Source: Animals (Basel). 2026 Jun 8;16(12):1774. doi: 10.3390/ani16121774 (PMC13295260; doi:10.3390/ani16121774)
Supplement: Supplementary file 1 [file animals-16-01774-s001.zip › animals-4327275-supplementary.pdf]

Supplemental materials

Table S1: Synergistic antibacterial effects of AMPs and antibiotics in mammals.

AMP	Antibiotic	Target pathogens	FICI	References
Bip-P-113	Vancomycin	*E. faecium* (BCRC 15B0132)	0.38	[1]
Bip-P-113	Vancomycin	*S. aureus* (VISA 01, 02, 03)	0.5	[1]
Bip-P-113	Vancomycin	*E. coli* (ATCC 25922)	0.38	[1]
CATH-1	Erythromycin	*S. aureus*	0.375	[2]
CATH-1	Ampicillin	*S. aureus*	0.5	[2]
CATH-1	Erythromycin	*S. enteritidis*	0.281	[2]
CATH-1	Erythromycin	*E. coli*	0.125	[2]
CATH-3	Erythromycin	*S. aureus*	0.281	[2]
CATH-3	Ampicillin	*S. aureus*	0.281	[2]
CATH-3	Erythromycin	*S. enteritidis*	0.258	[2]
CATH-3	Ampicillin	*S. enteritidis*	0.188	[2]
CATH-3	Erythromycin	*E. coli*	0.141	[2]
Citropin 1.1	Polymyxin E	*P. aeruginosa*	0.312	[3]
Citropin 1.1	Clarithromycin	*R. equi*	0.312	[4]
Citropin 1.1	Doxycycline	*R. equi*	0.385	[4]
Citropin 1.1	Rifampicin	*R. equi*	0.385	[4]
CLS001	Vancomycin	*S. aureus* (SAU2)	0.25	[5]
CLS001	Vancomycin	*P. aeruginosa* (PAER10)	0.28	[5]
CLS001	Azithromycin	*S. aureus* (SAU7)	0.19	[5]
CLS001	Azithromycin	*A. baumannii* (ABA3)	0.5	[5]
CLS001	Amoxicillin	*S. aureus* (SAU2)	0.5	[5]
CLS001	Amoxicillin	*P. aeruginosa* (PAER11)	0.5	[5]
CLS001	Amoxicillin	*A. baumannii* (ABA1)	0.5	[5]
Dip-P-113	Vancomycin	*E. faecium* (BCRC 15B0132)	0.5	[1]
Dip-P-113	Vancomycin	*S. aureus* (VRSA 02)	0.5	[1]
Dip-P-113	Vancomycin	*E. coli* (ATCC 25922)	0.5	[1]
DP-23	Ampicillin	*S. typhimurium* (MTCC98)	0.385	[6]
DP-23	Tetracycline	*E. coli* (ATCC25922)	0.511	[6]
DP-23	Tetracycline	*S. typhimurium* (MTCC98)	0.385	[6]
DP-23	Gentamicin	*E. coli* (IGMC/SRN/21)	0.25	[6]
DP7	Vancomycin	*S. aureus* (SAU2)	0.52	[5]
DP7	Vancomycin	*P. aeruginosa* (PAER11)	0.25	[5]
DP7	Vancomycin	*E. coli* (ECO3)	0.5	[5]
DP7	Vancomycin	*A. baumannii* (ABA1)	0.38	[5]
DP7	Azithromycin	*S. aureus* (SAU2)	0.01	[5]
DP7	Azithromycin	*P. aeruginosa* (PAER1)	0.04	[5]
DP7	Azithromycin	*E. coli* (ECO2)	0.5	[5]

AMP	Antibiotic	Target pathogens	FICI	References
DP7	Azithromycin	*A. baumannii* (ABA3)	0.31	[5]
DP7	Amoxicillin	*S. aureus* (SAU7)	0.5	[5]
DP7	Amoxicillin	*A. baumannii* (ABA1)	0.25	[5]
FLIP7	Meropenem	*A. baumannii* (28)	0.393	[7]
FLIP7	Cefotaxime	*E. coli* (ATCC 25922)	0.415	[7]
FLIP7	Chloramphenicol	*S. aureus* (203)	0.418	[7]
FLIP7	Polymyxin B	*E. coli* (ATCC 25922)	0.339	[7]
HE10α	Streptomycin	*E. coli* (XL-8 blue)	0.3	[8]
HE2α	Ampicillin	*E. coli* (XL-1 blue)	0.3	[8]
HE2α	Tetracycline	*E. coli* (XL-9 blue)	0.3	[8]
HE2β2	Ampicillin	*E. coli* (XL-10 blue)	0.3	[8]
HE2β2	Chloramphenicol	*E. coli* (XL-11 blue)	0.3	[8]
HE2β2	Carbenicillin	*E. coli* (XL-12 blue)	0.2	[8]
HE2β2	Ciprofloxacin	*E. coli* (XL-13 blue)	0.1	[8]
HE2β2	Doxycycline	*E. coli* (XL-14 blue)	0.2	[8]
HE2β2	Gentamicin	*E. coli* (XL-15 blue)	0.2	[8]
HE2β2	Kanamycin	*E. coli* (XL-16 blue)	0.2	[8]
HE2β2	Rifampicin	*E. coli* (XL-17 blue)	0.2	[8]
HE2β2	Streptomycin	*E. coli* (XL-18 blue)	0.1	[8]
HE2β2	Tetracycline	*E. coli* (XL-19 blue)	0.4	[8]
HE3α	Chloramphenicol	*E. coli* (XL-1 blue)	0.3	[8]
HE4α	Carbenicillin	*E. coli* (XL-2 blue)	0.3	[8]
HE5α	Ciprofloxacin	*E. coli* (XL-3 blue)	0.3	[8]
HE6α	Doxycycline	*E. coli* (XL-4 blue)	0.2	[8]
HE7α	Gentamicin	*E. coli* (XL-5 blue)	0.3	[8]
HE8α	Kanamycin	*E. coli* (XL-6 blue)	0.3	[8]
HE9α	Rifampicin	*E. coli* (XL-7 blue)	0.3	[8]
LL-37	Polymyxin B	*E. coli* K-12 (MG1655)	0.37	[9]
LL-37	Polymyxin B	*P. aeruginosa* (PAO1)	0.31	[9]
LL-37	Polymyxin B	*P. aeruginosa* (PAO1)	0.31	[9]
LL-37	Polymyxin B	*E. coli* (MG1655)	0.37	[9]
LL-37	Ciprofloxacin	*E. coli* (MG1655)	0.5	[9]
LP-23	Tetracycline	*S. aureus* (MTCC3160)	0.314	[6]
LP-23	Tetracycline	*S. typhimurium* (MTCC98)	0.384	[6]
LP-23	Gentamicin	*E. coli* (IGMC/SRN/21)	0.145	[6]
Magainin II	Imipenem	*P. aeruginosa*	0.385	[3]
Magainin II	Imipenem	*S. aureus*	0.385	[3]
Magainin II	Ceftazidime	*P. aeruginosa*	0.312	[3]
Magainin II	Polymyxin E	*P. aeruginosa*	0.312	[3]
Melittin	penicillin	*S. aureus*	0.37	[10]
Melittin	penicillin	*S. aureus*	0.37	[10]
Nal-P-113	Vancomycin	*E. faecium* (BCRC 15B0132)	0.5	[1]
Nal-P-113	Vancomycin	*S. aureus* (VISA 03)	0.5	[1]

AMP	Antibiotic	Target pathogens	FICI	References
Nal-P-113	Vancomycin	*E. coli* (ATCC 25922)	0.38	[1]
Nisin	Colistin	*A. baumannii* (ATCC 19606)	0.5	[11]
Nisin	Tobramycin	*A. baumannii* (XDR2)	0.325	[11]
Nisin	Ciprofloxacin	*S. aureus* (ATCC 27853)	0.5	[11]
Nisin	Doripenem	*A. baumannii* (ATCC 19606)	0.312	[11]
P10	Ceftazidime	*A. baumannii* (XDR3)	0.187	[11]
P10	Doripenem	*A. baumannii* (XDR1)	0.375	[11]
PMAP-36	Erythromycin	*S. aureus*	0.313	[2]
PMAP-36	Ampicillin	*S. aureus*	0.5	[2]
PMAP-36	Erythromycin	*S. enteritidis*	0.281	[2]
PMAP-36	Ampicillin	*S. enteritidis*	0.313	[2]
PMAP-36	Erythromycin	*E. coli*	0.254	[2]
SA4	Ampicillin	*E. coli* (ATCC25922)	0.385	[6]
SA4	Tetracycline	*S. typhimurium* (MTCC98)	0.252	[6]
SPO	Tetracycline	*E. coli* (ATCC25922)	0.386	[6]
SPO	Tetracycline	*S. typhimurium* (MTCC98)	0.511	[6]
SPO	Gentamicin	*E. coli* (IGMC/SRN/21)	0.187	[6]
Temporin A	Imipenem	*P. aeruginosa*	0.458	[3]
Temporin A	Imipenem	*S. aureus*	0.312	[3]
Temporin A	Ceftazidime	*P. aeruginosa*	0.385	[3]
Temporin A	Polymyxin E	*P. aeruginosa*	0.385	[3]
Temporin A	Linezolid	*S. aureus*	0.385	[3]

Synergy: FICI ≤ 0.5.

Table S2: Synergistic antibacterial effects of AMPs and polysaccharides in mammals.

AMP	Polysaccharide	Formation Mode of Nanoparticles	Synergy Mechanism	References
CATH–FLA	Chitosan	Spray drying to form microparticles encapsulating CATH–FLA.	Chitosan protects CATH–FLA from gastric degradation; sustained intestinal release enhances bioactivity.	[12]
CM11	Hyaluronic acid (HA)	Ionic gelation (CS NPs coated with HA via EDC coupling)	Targeted delivery via CD44 receptor; pH-sensitive drug release in tumor microenvironment	[13]
CR2109	Sodium alginate & pectin	Spray-dried alginate-pectin microparticles incorporating self-assembled CR2109 and resveratrol.	AMP enhances structural integrity of alginate matrix; sustained release in intestines.	[14]
EPL	Hyaluronic Acid (HA) & Polyvinyl Alcohol (PVA)	Rapidly dissolving and detachable HA-tipped PVA microneedles loaded with EPL	HA tips enable rapid drug release; PVA substrate provides mechanical support and detachability for convenient application	[15]
HHC36	Carboxymethyl chitosan (CMCS) & sodium alginate (SA)	Co-delivery via CMCS/SA hydrogel with AgNPs conjugation (Ag-H)	- HHC36 enhances penetration into dermis, targeting bacterial infection sites.<br>- AgNPs release Ag+ ions, disrupting bacterial proteins; HHC36 disrupts membranes.	[16]
HX-12C	Chitosan	Incorporated into chitosan films via physical mixing/solution casting.	Chitosan provides controlled release of HX-12C, enhancing antibacterial activity and stability.	[17]
LL37	Chitosan	Ionic gelation method between sodium tripolyphosphate (TPP) and chitosan.	Chitosan protects LL37 from degradation, controls release, and enhances membrane interaction.	[18]
LLKKK18	Hyaluronic Acid (HA)	Self-assembling HA nanogels encapsulating LLKKK18	Nanogels enhance stability, reduce cytotoxicity, and improve targeting to infection sites	[19]
NRC-07	Chitosan (CS)	Ionotropic gelation (CS-NPs loaded with NRC-07)	- CS-NPs enhance stability of NRC-07, protecting it from degradation.<br>- CS-NPs facilitate targeted delivery to bacterial/cancer cell membranes via electrostatic interactions.	[20]
Pexiganan	Sodium alginate & Chitosan	Pexiganan-sodium alginate nanoparticles gel-formed and combined with chitosan-cholesterol.	Enhanced membrane permeabilization, ROS generation, elevated lipid peroxidation in bacterial membranes.	[21]
Piscidin-1	Chitosan & β-glycerolphosphate disodium salt pentahydrate	Thermo-responsive chitosan hydrogel loaded with Piscidin-1 via β-glycerolphosphate cross-linking.	Chitosan provides sustained release of Piscidin-1, enhancing antibacterial activity against resistant bacteria.	[22]
Polymyxin B	Sodium alginate (SA)	Ionic crosslinking to form composite hydrogel	- The polysaccharide matrix promotes cell proliferation and migration, while AMPs	[23]

AMP	Polysaccharide	Formation Mode of Nanoparticles	Synergy Mechanism	References
and Bacitracin		nanoparticles	provide antibacterial protection, synergistically enhancing wound healing.	

Table S3: Synergistic antibacterial effects of AMPs and herbal extracts in mammals.

AMP	Herbal extracts	Target pathogens	FICI	Synergy mechanism	References
Nisin	Curcumin	*L. monocytogenes* (CICC 21633)<br>*B. subtilis* (CICC 10,275)<br>*S. aureus* (CICC 10,384)	ND	The encapsulation of curcumin within the Nisin-SSPS complex enhances its solubility and stability, with Nisin providing initial antimicrobial action and curcumin contributing sustained effects through controlled release from the nanoparticles; this encapsulation also protects curcumin from degradation, improving its thermal and storage stability, while enhancing its antioxidant properties to potentially reduce oxidative stress during infections	[24]
KN5	Thymol	*S. aureus* (ATCC 43300)	0.375	KN5 and thymol synergistically compromise cell structure integrity, cause leakage of intracellular substances, dissipate proton motive force, enhance ROS generation, and improve DNA binding affinity, leading to bacterial death.	[25]
Magainin II	Curcumin/Azithromycin (AZI)	*S. aureus* (ATCC29213)	ND	MagII-modified micelles enhance targeting to bacterial infection sites, release AZI and curcumin in response to acidic microenvironment, reduce bacterial resistance, and improve antibacterial efficacy through multiple mechanisms (e.g., ROS induction, biofilm disruption).	[26]
CF-14	Eugenol (EU) or Carvacrol (CAR)	*E*．*coli*<br>*S*．*aureus*	ND	The combined use of CF-14 with eugenol and carvacrol leads to increased bacterial cell death, particularly effective against *E. coli* due to improved membrane penetration and DNA damage; however, the effect is reduced against *S. aureus* due to membrane charge differences.	[27]
REDV	Eugenol	*E*．*coli* (ATCC 8739)<br>*S*．*aureus* (ATCC 6538)	ND	The REDV peptide, when conjugated with PCL and incorporated into electrospun membranes along with eugenol, exhibits a dual-function synergistic effect; REDV enhances the adhesion and proliferation of vascular endothelial cells while eugenol provides significant antibacterial activity against both *E. coli* and *S. aureus*, with the electrospun membrane structure facilitating controlled release of eugenol for sustained antimicrobial effects.	[28]
Pexiganan	Trans-chalcone (TC)	*S*．*aureus* (ATCC 25923)<br>*S*．*aureus* (ATCC 43300)<br>*E. coli* (ATCC 25922)<br>*P. aeruginosa* (ATCC 27853)	≤0.5	When combined with PEX, TC PNPs exhibit synergistic antibacterial activity against all tested bacteria by enhancing cell membrane permeability and inhibiting bacterial Sortase A enzyme activity, reducing the effective dose and toxicity of each component.	[29]
Pexiganan	Berberine (BR)	*S. aureus* (ATCC 25923)<br>*S. aureus* (ATCC 43300)	≤0.5	When combined with PEX, BR PNPs exhibit synergistic antibacterial activity against MSSA and MRSA by enhancing cell membrane permeability and inhibiting bacterial Sortase A enzyme activity, reducing the effective dose of each component.	[29]
LL37	Curcumin (Cur)	*E. coli*	ND	The combination of LL37 and curcumin encapsulated in chitosan nanoparticles exhibits	[30]

				synergistic antibacterial effects by enhancing membrane disruption and inhibiting bacterial growth, improving intestinal microflora and immune response.	
AS-48	Carvacrol, Geraniol, Eugenol, Terpineol, Caffeic acid, p-Coumaric acid, Citral, Hydrocinnamic acid	*S. aureus (CECT 976)*	ND	The antimicrobial activity of AS-48 against *S. aureus* is significantly potentiated when used in combination with various phenolic compounds, exhibiting additive or synergistic effects that depend on the type of phenolic compound and the food matrix.	[31]
Nisin	Avocado seed extract	*L. innocua* (ATCC 33090)	ND	Although the avocado seed extract alone does not exhibit antimicrobial activity, its combination with nisin shows a synergistic effect, enhancing the antimicrobial response against *L. innocua*.	[32]

ND：Not deceted; Synergy: FICI ≤ 0.5.

Table S4 Synergistic antibacterial effects of AMPs and AMPs in mammals.

AMP1	AMP2	Target pathogens	FICI	Synergy mechanism	References
Abaecin	Hymenoptaecin	*E. coli* (JM83)	ND	Abaecin potentiates the activity of hymenoptaecin by interacting with bacterial chaperone DnaK after the bacterial membrane is compromised by hymenoptaecin.	[33]
Amylin	Aβ42	*S. Typhimurium* (SL1344)<br>*S. aureus* (ATCC25923)	ND	Amylin and Aβ42 exhibit synergistic antimicrobial activity by forming fibrils that entrap and neutralize microbes, enhancing bacterial killing. Additionally, the combination may induce microbial membrane disruption and increase intracellular ROS levels, leading to enhanced bactericidal effects.	[34]
AW1	AW2	*K. pneumoniae* (ATCC43863)<br>*S. aureus* (CMCC26003)	0.3125<br>0.375	AW2 enhances the antibacterial activity of AW1 by facilitating its cellular uptake and intracellular localization, where AW2 binds to bacterial genomic DNA, inhibiting replication and transcription. Additionally, the combination triggers ROS generation, which contributes to bacterial oxidative stress and death.	[35]
BHL-bombinin	Bombinin HL	*S. aureus* (NCTC10788)	0.375	ND	[36]
BHL-bombinin	Bombinin HD	*S. aureus* (NCTC10788)	0.375	ND	[36]
Bombinin	Feleucin-BV1	*S. aureus* (ATCC25923)	0.5	ND	[37]
CA(1–7)M(2–9)NH2	Temporin A	*S. aureus* (357426)<br>*S. aureus* (355872)<br>*S. aureus* (348839)<br>*S. aureus* (ATCC43300)	0.25<br>0.38<br>0.5<br>0.26	ND	[38]

AMP1	AMP2	Target pathogens	FICI	Synergy mechanism	References
CA(1–7)M(2–9)NH2	Citropin 1.1	*S. aureus* (357426)	0.5	ND	[38]
		S. aureus (355872)	0.48		
		S. aureus (348839)	0.5		
		S. aureus (ATCC43300)	0.5		
CA(1–7)M(2–9)NH2	Pal-KGK-NH2	*S. aureus* (348839)	0.5	ND	[38]
		S. aureus (ATCC43300)	0.5		
Cec6	Def4	*M. luteus*	0.41	ND	[39]
CP10A	Sub5-01	*E. coli* (25922)	<0.5	ND	[40]
CP10A	Mag2-01	*E. coli* (25922)	<0.5	ND	[40]
		S. aureus (MRSA 43300)	<0.5		
		E. faecium (700221 VRE)	<0.5		
CP10A	OV3-01	*E. coli* (25922)	<0.5	ND	[40]
		E. faecium (700221 VRE)	<0.5		
CP10A	Sub5	*E. coli* (ATCC 25922)	<0.5	Multiple mechanisms including membrane disruption, DNA damage, and increased ROS production.	[41]
CP10A	Cu-Sub5-1	*E. coli* (ATCC 25922)	<0.5	Multiple mechanisms including membrane disruption, DNA damage, and increased ROS production.	[41]
		S. aureus (ATCC 43300)	<0.5		
CP10A	Cu-Sub5-2	*E. coli* (ATCC 25922)	<0.5	Multiple mechanisms including membrane disruption, DNA damage, and increased ROS production.	[41]
		S. aureus (ATCC 43300)	<0.5		
CRAMP	Api88	*E. coli* (ATCC 25922)	0.36	ND	[42]
CRAMP	Onc110	*E. coli* (ATCC 25922)	0.3	ND	[42]
		A. baumannii (DSM 30008)	0.44		
		P. aeruginosa (DSM 9644)	0.41		
Defensin 5	Defensin 6	*E. coli* (ATCC 25922)	NA	Defensin 6 specifically and synergistically enhances Defensin -5-induced IL-8	[43]

AMP1	AMP2	Target pathogens	FICI	Synergy mechanism	References
		S. aureus (ATCC 29213)		secretion without altering its antibacterial activity. HD-6 also negates the HD-5-induced decrease in transepithelial electrical resistance.	
Dode	MAP-28	*E. coli* (ML-35p)<br>*S. aureus* (209P)	0.156<br><0.188	ND	[44]
Gallidermin	DCD-1	*S. aureus* (USA300)	<0.5	ND	[45]
Gallidermin	DCD-1L	*S. aureus* (USA300)	<0.5	ND	[45]
Gallinacin 7	Gallinacin 9	*S. enteriditis*	ND	ND	[46]
GNCPs	CAP11	*E. coli* (NIHJ JC-2)<br>*S. aureus* (NIHJ JC-1)	ND	GNCPs synergizes with CAP11 to disrupt target cell membranes, enhancing antibacterial activity.	[47]
HNP-1	LL-37	*E. coli* (NIHJ JC-2)<br>*S. aureus* (NIHJ JC-1)	ND	HNP-1 synergizes with LL-37 to disrupt target cell membranes, enhancing antibacterial activity.	[47]
Indolicidin	Protegrin-1	*E. coli* (HB101)<br>*P. aeruginosa* (PAO1)	0.25<br>0.25	ND	[48]
Indolicidin	Bactenecin	*E. coli* (HB101)	0.5	ND	[48]
LL-37	Protegrin-1	*E. coli* (HB101)<br>*P. aeruginosa* (PAO1 strain H103)<br>*E. faecalis* (ATCC 29212)	0.31<br>0.31<br>0.32	ND	[48]
LL-37	β-defensin 2	*S. aureus* (isolated)<br>*Group B Streptococcus* (COHI type III)<br>*P. gingivalis* (Pgm6/Pgm7-deficient strain)	ND	ND	[49-51]
LL-37	Gallidermin	*S. aureus* (USA300)	<0.5	ND	[45]
LL-37	β-defensin 1	*P. gingivalis* (Pgm6/Pgm7-deficient strain)	ND	ND	[51]
LL-37	β-defensin 3	*P. gingivalis* (Pgm6/Pgm7-deficient	ND	ND	[51]

AMP1	AMP2	Target pathogens	FICI	Synergy mechanism	References
		strain)			
MAG2	PGLa	*E. coli* (DH5α)<br>*E. helveticus* (DSM18396)<br>*K. rhizophila* (DSM348)	0.3<br>0.3<br>0.3	Synergistic activity likely arises from specific molecular interactions between MAG2 and PGLa, possibly involving heterodimer formation that enhances membrane permeabilization and antimicrobial effects.	[52]
Magainin 2	Tachyplesin 1	*Escherichia coli* (NA)	ND	Magainin 2 and Tachyplesin 1 co-aggregate in solution to form smaller, more active hetero-oligomers compared to individual peptides. These hetero-oligomers enhance recognition and disruption of bacterial membranes, leading to increased antibacterial activity.	[53]
Nisin	Colistin	*A. baumannii* (ATCC 19606)<br>*A. baumannii* (XD-R2)<br>*S. aureus* (ATCC 27853)<br>*S. aureus* (col 1)<br>*S. aureus* (col 2)<br>*S. aureus* (col 5)	0.312<br>0.5<br>0.375<br>0.5<br>0.375<br>0.5	ND	[11]
P10	Nisin	*A. baumannii* (ATCC 19606)<br>*S. aureus* (ATCC 27853)	0.5<br>0.5	ND	[11]
PAF	PAF26	*P. digitatum* (CECT20796)<br>*A. niger* (CBS 120.49)	ND	ND	[54]
PAF118	PAF112	*P. digitatum* (CECT20796)	ND	ND	[54]
PAF118	PAF26	*P. digitatum* (CECT20796)	ND	ND	[54]
PAF26	PAF112	*P. digitatum* (CECT20796)	ND	ND	[54]
PAF26	PAF116	*P. digitatum* (CECT20796)	ND	ND	[54]
Protegrin-1	Lysozyme	*E. faecalis* (ATCC 29212)	0.31	ND	[48]
Protonectin	Protonectin (1–6)	*E. coli* (ATCC 25922)<br>*B. subtilis* (CCT2576)	ND	ND	[55]

AMP1	AMP2	Target pathogens	FICI	Synergy mechanism	References
		P. aeruginosa (ATCC 15442)<br>*S. aureus* (ATCC 6538)			
R44K S	V31K S	*S. aureus* (209P)	0.31	ND	[56]
R44K S	R23F S	*S. aureus* (ATCC 43300)	0.5	ND	[56]
SynSaf-P8	SynSaf-P96	*E. coli* (NCTC12923)<br>*A. baumannii* (AYE)	0.25<br>0.5	SynSaf-P8 enhances the pore-forming capability of SynSaf-P96, leading to increased membrane disruption and bacterial killing.	[57]
UyCT3	UyCT5	*E. coli* (ATCC 25922)	ND	UUyCT3 and UyCT5 synergize by enhancing membrane disruption through cooperative interaction	[58]
WW-185	WOW	*E. coli* (ATCC 25250)<br>*E. coli* (ATCC BAA-2219)<br>*S. aureus* (ATCC 25923)<br>*S. aureus* (ATCC BAA-2313)	0.1006<br>0.303<br>0.02712<br>0.327	ND	[59]
β-defensin-1	PG-3	*E. coli* (ATCC 35218)<br>*S. typhimurium* (DT104)	ND	ND	[60]
β-defensin-1	PR-39	*E. coli* (ATCC 35218)	ND	ND	[60]

Synergy: FICI ≤ 0.5.

References:

1. Wu, C.L.; Hsueh, J.Y.; Yip, B.S.; Chih, Y.H.; Peng, K.L.; Cheng, J.W. Antimicrobial peptides display strong synergy with vancomycin against vancomycin-resistant *E. faecium*, *S. aureus*, and Wild-Type *E. coli*. *Int J Mol Sci* **2020**, *21*.
2. Lu, Y.; Tian, H.; Chen, R.; Liu, Q.; Jia, K.; Hu, D.L.; Chen, H.; Ye, C.; Peng, L.; Fang, R. Synergistic antimicrobial effect of antimicrobial peptides CATH-1, CATH-3, and PMAP-36 with erythromycin against bacterial pathogens. *Front Microbiol* **2022**, *13*, 953720.
3. Giacometti, A.; Cirioni, O.; Kamysz, W.; Silvestri, C.; Licci, A.; Riva, A.; Łukasiak, J.; Scalise, G. *In vitro* activity of amphibian peptides alone and in combination with antimicrobial agents against multidrug-resistant pathogens isolated from surgical wound infection. *Peptides* **2005**, *26*, 2111-2116.
4. Giacometti, A.; Cirioni, O.; Kamysz, W.; Silvestri, C.; Del Prete, M.S.; Licci, A.; D'Amato, G.; Lukasiak, J.; Scalise, G. *In vitro* activity of citropin 1.1 alone and in combination with clinically used antimicrobial agents against *Rhodococcus equi*. *J Antimicrob Chemother* **2005**, *56*, 410-412.
5. Wu, X.; Li, Z.; Li, X.; Tian, Y.; Fan, Y.; Yu, C.; Zhou, B.; Liu, Y.; Xiang, R.; Yang, L. Synergistic effects of antimicrobial peptide DP7 combined with antibiotics against multidrug-resistant bacteria. *Drug Des Devel Ther* **2017**, *11*, 939-946.
6. Sharma, L.; Bisht, G.S. Synergistic effects of short peptides and antibiotics against bacterial and fungal strains. *J Pept Sci* **2023**, *29*, e3446.
7. Chernysh, S.; Gordya, N.; Tulin, D.; Yakovlev, A. Biofilm infections between Scylla and Charybdis: Interplay of host antimicrobial peptides and antibiotics. *Infect Drug Resist* **2018**, *11*, 501-514.
8. Yenugu, S.; Narmadha, G. The human male reproductive tract antimicrobial peptides of the HE2 family exhibit potent synergy with standard antibiotics. *J Pept Sci* **2010**, *16*, 337-341.
9. Ridyard, K.E.; Elsawy, M.; Mattrasingh, D.; Klein, D.; Strehmel, J.; Beaulieu, C.; Wong, A.; Overhage, J. Synergy between human peptide LL-37 and polymyxin B against planktonic and biofilm cells of *Escherichia coli* and *Pseudomonas aeruginosa*. *Antibiotics (Basel)* **2023**, *12*, 389.
10. Jalalifar, S.; Razavi, S.; Mirzaei, R.; Irajian, G.; Pooshang Bagheri, K. A hope for ineffective antibiotics to return to treatment: investigating the anti-biofilm potential of melittin alone and in combination with penicillin and oxacillin against multidrug resistant-MRSA and -VRSA. *Front Microbiol* **2023**, *14*, 1269392.
11. Jahangiri, A.; Neshani, A.; Mirhosseini, S.A.; Ghazvini, K.; Zare, H.; Sedighian, H. Synergistic effect of two antimicrobial peptides, Nisin and P10 with conventional antibiotics against extensively drug-resistant *Acinetobacter baumannii* and colistin-resistant *Pseudomonas aeruginosa* isolates. *Microb Pathog* **2021**, *150*, 104700.
12. González-Chavarría, I.; Roa, F.J.; Sandoval, F.; Muñoz-Flores, C.; Kappes, T.; Acosta, J.; Bertinat, R.; Altamirano, C.; Valenzuela, A.; Sánchez, O., et al. Chitosan microparticles enhance the intestinal release and immune response of an immune stimulant peptide in *Oncorhynchus mykiss*. *Int J Mol Sci* **2023**, *24*, 3390.
13. Taghipour-Sabzevar, V.; Sharifi, T.; Bagheri-Khoulenjani, S.; Goodarzi, V.; Kooshki, H.; Halabian, R.; Moosazadeh Moghaddam, M. Targeted delivery of a short antimicrobial peptide against CD44-overexpressing tumor cells using hyaluronic acid-coated chitosan nanoparticles:

An *in vitro* study. *J. Nanoparticle Res.* **2020**, *22*, 99.
14. Roque-Borda, C.A.; Chávez-Morán, M.R.; Primo, L.; Montesinos, J.; Cardoso, V.M.B.; Saraiva, M.M.S.; Marcos, C.M.; Chorilli, M.; Albericio, F.; de la Torreh, B.G., et al. Alginate-pectin microparticles embedding self-assembling antimicrobial peptides and resveratrol for antimicrobial and anti-inflammatory applications. *Food Hydrocoll* **2025**, *167*, 111454.
15. Zheng, H.; Liu, N.; Zhou, T.; Wang, Y.; Shi, L.; Wang, P.; Li, P. Rapidly dissolving and detachable microneedles loaded with antimicrobial peptides for acne vulgaris treatment. *J Mater Chem B* **2025**, *13*, 15077-15089.
16. Liu, H.; Zhan, J.; Lin, R.; Yin, Y.; Ren, L. Strong infiltrative HHC36 antimicrobial peptide/silver nanoparticles-loaded carboxymethyl chitosan/sodium alginate hydrogel for acne vulgaris therapy. *Nanotechnology* **2023**, *34*, 495101.
17. Luo, X.F.; Peng, Y.F.; Qin, Z.D.; Tang, W.F.; Duns, G.J.; Dessie, W.; He, N.Y.; Tan, Y.M. Chitosan-based packaging films with an integrated antimicrobial peptide: Characterization, *in vitro* release and application to fresh pork preservation. *Int J Biol Macromol* **2023**, *231*.
18. Rashki, S.; Safardoust-Hojaghan, H.; Mirzaei, H.; Abdulsahib, W.K.; Mahdi, M.A.; Salavati-Niasari, M.; Khaledi, A.; Khorshidi, A.; Mousavi, S.G.A. Delivery LL37 by chitosan nanoparticles for enhanced antibacterial and antibiofilm efficacy. *Carbohydr Polym* **2022**, *291*, 119634.
19. Silva, J.P.; Gonçalves, C.; Costa, C.; Sousa, J.; Silva-Gomes, R.; Castro, A.G.; Pedrosa, J.; Appelberg, R.; Gama, F.M. Delivery of LLKKK18 loaded into self-assembling hyaluronic acid nanogel for tuberculosis treatment. *J Control Release* **2016**, *235*, 112-124.
20. Turky, N.O.; Abdelmonem, N.A.; Tammam, S.N.; Gad, M.Z.; Breitinger, H.G.; Breitinger, U. Antibacterial and *in vitro* anticancer activities of the antimicrobial peptide NRC-07 encapsulated in chitosan nanoparticles. *J Pept Sci* **2024**, *30*, e3550.
21. Saha, S.; Kar, R.; Sikder, K.; Manna, D.; Pal, R.R.; Chakraborti, S.; Khan, A.H.; Barman, S.; Maity, A.R.; Basu, A. Deciphering the inhibitory mechanism of antimicrobial peptide pexiganan conjugated with sodium-alginate chitosan-cholesterol nanoparticle against the opportunistic pathogen *Acinetobacter baumannii*. *J. Drug Deliv. Sci. Technol* **2024**, *101*, 106305.
22. Rezaei, N.; Hamidabadi, H.G.; Khosravimelal, S.; Zahiri, M.; Ahovan, Z.A.; Bojnordi, M.N.; Eftekhari, B.S.; Hashemi, A.; Ganji, F.; Darabi, S., et al. Antimicrobial peptides-loaded smart chitosan hydrogel: Release behavior and antibacterial potential against antibiotic resistant clinical isolates. *Int J Biol Macromol* **2020**, *164*, 855-862.
23. Feng, X.; Zhang, X.; Li, S.; Zheng, Y.; Shi, X.; Li, F.; Guo, S.; Yang, J. Preparation of aminated fish scale collagen and oxidized sodium alginate hybrid hydrogel for enhanced full-thickness wound healing. *Int J Biol Macromol* **2020**, *164*, 626-637.
24. Luo, L.; Wu, Y.; Liu, C.; Zou, Y.; Huang, L.; Liang, Y.; Ren, J.; Liu, Y.; Lin, Q. Elaboration and characterization of curcumin-loaded soy soluble polysaccharide (SSPS)-based nanocarriers mediated by antimicrobial peptide nisin. *Food Chem* **2021**, *336*, 127669.
25. Ma, M.; Song, J.; Wu, Y.; Hu, Y.; Xiao, Y.; Liu, J.; Wu, C.; Hu, Z.; Zeng, B. Synergistic effects of antimicrobial peptide KRR-N1-5W6L and thymol on the inactivation of methicillin-resistant *Staphylococcus aureus* in chilled beef. *Int J Food Microbiol* **2026**, *445*, 111503.
26. Zhang, L.; Guo, R.-b.; Liu, Y.; Kong, L.; Zang, J.; Zhang, Z.-x.; Wang, J.-h.; Chen, M.-h.; Liu, M.; Yu, Y. Therapeutic effect of pH responsive Magainin II modified azithromycin plus curcumin micelles in different depth models of MRSA infection. *Sci Rep* **2025**, *15*, 7383.

27. Majeed, U.; Majeed, H.; Shafi, A.; Liu, X.; Ye, J.; Wang, Y.; Xue, W.; Luo, Y.; Yue, T. DNA binding peptide CF-14 enhances bactericidal efficacy of eugenol/carvacrol nanoparticles to *Escherichia coli*. *Food Chem* **2023**, *429*, 136861.
28. Li, Z.; Zhou, P.; Zhou, F.; Zhao, Y.; Ren, L.; Yuan, X. Antimicrobial eugenol-loaded electrospun membranes of poly(ε-caprolactone)/gelatin incorporated with REDV for vascular graft applications. *Colloids Surf B Biointerfaces* **2018**, *162*, 335-344.
29. Alharthi, S.; Popat, A.; Ziora, Z.M.; Moyle, P.M. Sortase A inhibitor protein nanoparticle formulations demonstrate antibacterial synergy when combined with antimicrobial peptides. *Molecules* **2023**, *28*, 2114.
30. Wan, X.; Liu, L.; Ding, L.; Zhu, Z. Fabrication of bio-engineered chitosan nanoformulations to inhibition of bacterial infection and to improve therapeutic potential of intestinal microflora, intestinal morphology, and immune response in infection induced rat model. *Drug Deliv* **2022**, *29*, 2002-2016.
31. Grande, M.J.; López, R.L.; Abriouel, H.; Valdivia, E.; Ben Omar, N.; Maqueda, M.; Martínez-Cañamero, M.; Gálvez, A. Treatment of vegetable sauces with enterocin AS-48 alone or in combination with phenolic compounds to inhibit proliferation of *Staphylococcus aureus*. *J Food Prot* **2007**, *70*, 405-411.
32. Calderón-Oliver, M.; Escalona-Buendía, H.B.; Medina-Campos, O.N.; Pedraza-Chaverri, J.; Pedroza-Islas, R.; Ponce-Alquicira, E. Optimization of the antioxidant and antimicrobial response of the combined effect of nisin and avocado byproducts. *LWT-FOOD SCI TECHNOL* **2016**, *65*, 46-52.
33. Rahnamaeian, M.; Cytryńska, M.; Zdybicka-Barabas, A.; Dobslaff, K.; Wiesner, J.; Twyman, R.M.; Zuchner, T.; Sadd, B.M.; Regoes, R.R.; Schmid-Hempel, P., et al. Insect antimicrobial peptides show potentiating functional interactions against Gram-negative bacteria. *Proc Biol Sci* **2015**, *282*, 20150293.
34. Vijaya Kumar, D.K.; Mitchell, T.A.; Tailor, B.A.; Moir, A.P.; Navalpur Shanmugam, N.K.; Eimer, W.A.; Ghelichi, J.; Choi, S.H.; Su, C.; Rodriguez, A.S., et al. Human amylin is a potent antimicrobial peptide that exhibits antimicrobial synergism with the amyloid beta protein. *Alzheimers Dement* **2025**, *21*, e70490.
35. Ye, Z.; Fu, L.; Li, S.; Chen, Z.; Ouyang, J.; Shang, X.; Liu, Y.; Gao, L.; Wang, Y. Synergistic collaboration between AMPs and non-direct antimicrobial cationic peptides. *Nat Commun* **2024**, *15*, 7319.
36. Xiang, J.; Zhou, M.; Wu, Y.; Chen, T.; Shaw, C.; Wang, L. The synergistic antimicrobial effects of novel bombinin and bombinin H peptides from the skin secretion of *Bombina orientalis*. *Biosci Rep* **2017**, *37*.
37. Bai, B.; Hou, X.; Wang, L.; Ge, L.; Luo, Y.; Ma, C.; Zhou, M.; Duan, J.; Chen, T.; Shaw, C. Feleucins: novel bombinin precursor-encoded nonapeptide amides from the skin secretion of *Bombina variegata*. *Biomed Res Int* **2014**, *2014*, 671362.
38. Ciandrini, E.; Morroni, G.; Cirioni, O.; Kamysz, W.; Kamysz, E.; Brescini, L.; Baffone, W.; Campana, R. Synergistic combinations of antimicrobial peptides against biofilms of methicillin-resistant *Staphylococcus aureus* (MRSA) on polystyrene and medical devices. *J Glob Antimicrob Resist* **2020**, *21*, 203-210.
39. Pöppel, A.K.; Vogel, H.; Wiesner, J.; Vilcinskas, A. Antimicrobial peptides expressed in medicinal maggots of the blow fly *Lucilia sericata* show combinatorial activity against bacteria.

Antimicrob Agents Chemother **2015**, *59*, 2508-2514.
40. Greve, J.M.; Cowan, J.A. Activity and synergy of Cu-ATCUN antimicrobial peptides. *Int J Mol Sci* **2022**, *23*, 14151.
41. Thompson, Z.; Greve, J.M.; Cowan, J.A. Enhanced synergism and mechanism of action studies of synthetic antimicrobial metallopeptides. *ChemMedChem* **2021**, *16*, 2112-2120.
42. Knappe, D.; Kabankov, N.; Herth, N.; Hoffmann, R. Insect-derived short proline-rich and murine cathelicidin-related antimicrobial peptides act synergistically on Gram-negative bacteria *in vitro*. *Future Med Chem* **2016**, *8*, 1035-1045.
43. Zhao, A.; Lu, W.; de Leeuw, E. Functional synergism of human defensin 5 and human defensin 6. *Biochem Biophys Res Commun* **2015**, *467*, 967-972.
44. Bolosov, I.A.; Panteleev, P.V.; Sychev, S.V.; Sukhanov, S.V.; Mironov, P.A.; Myshkin, M.Y.; Shenkarev, Z.O.; Ovchinnikova, T.V. Dodecapeptide cathelicidins of cetartiodactyla: Structure, mechanism of antimicrobial action, and synergistic interaction with other cathelicidins. *Front Microbiol* **2021**, *12*, 725526.
45. Bitschar, K.; Sauer, B.; Focken, J.; Dehmer, H.; Moos, S.; Konnerth, M.; Schilling, N.A.; Grond, S.; Kalbacher, H.; Kurschus, F.C., et al. Lugdunin amplifies innate immune responses in the skin in synergy with host- and microbiota-derived factors. *Nat Commun* **2019**, *10*, 2730.
46. Milona, P.; Townes, C.L.; Bevan, R.M.; Hall, J. The chicken host peptides, gallinacins 4, 7, and 9 have antimicrobial activity against *Salmonella serovars*. *Biochem Biophys Res Commun* **2007**, *356*, 169-174.
47. Nagaoka, I.; Hirota, S.; Yomogida, S.; Ohwada, A.; Hirata, M. Synergistic actions of antibacterial neutrophil defensins and cathelicidins. *Inflamm Res* **2000**, *49*, 73-79.
48. Yan, H.; Hancock, R.E. Synergistic interactions between mammalian antimicrobial defense peptides. *Antimicrob Agents Chemother* **2001**, *45*, 1558-1560.
49. Ong, P.Y.; Ohtake, T.; Brandt, C.; Strickland, I.; Boguniewicz, M.; Ganz, T.; Gallo, R.L.; Leung, D.Y. Endogenous antimicrobial peptides and skin infections in atopic dermatitis. *N Engl J Med* **2002**, *347*, 1151-1160.
50. Dorschner, R.A.; Lin, K.H.; Murakami, M.; Gallo, R.L. Neonatal skin in mice and humans expresses increased levels of antimicrobial peptides: innate immunity during development of the adaptive response. *Pediatr Res* **2003**, *53*, 566-572.
51. Horie, T.; Inomata, M.; Into, T. OmpA-Like proteins of porphyromonas gingivalis mediate resistance to the antimicrobial peptide LL-37. *J Pathog* **2018**, *2018*, 2068435.
52. Strandberg, E.; Zerweck, J.; Horn, D.; Pritz, G.; Berditsch, M.; Bürck, J.; Wadhwani, P.; Ulrich, A.S. Influence of hydrophobic residues on the activity of the antimicrobial peptide magainin 2 and its synergy with PGLa. *J Pept Sci* **2015**, *21*, 436-445.
53. Remington, J.M.; Liao, C.; Sharafi, M.; Ste Marie, E.J.; Ferrell, J.B.; Hondal, R.J.; Wargo, M.J.; Schneebeli, S.T.; Li, J. Aggregation state of synergistic antimicrobial peptides. *J Phys Chem Lett* **2020**, *11*, 9501-9506.
54. Garrigues, S.; Gandía, M.; Borics, A.; Marx, F.; Manzanares, P.; Marcos, J.F. Mapping and identification of antifungal peptides in the putative antifungal protein AfpB from the filamentous fungus *Penicillium digitatum*. *Front Microbiol* **2017**, *8*, 592.
55. Baptista-Saidemberg, N.B.; Saidemberg, D.M.; de Souza, B.M.; César-Tognoli, L.M.; Ferreira, V.M.; Mendes, M.A.; Cabrera, M.P.; Ruggiero Neto, J.; Palma, M.S. Protonectin (1-6): a novel chemotactic peptide from the venom of the social wasp *Agelaia pallipes* pallipes. *Toxicon* **2010**,

56, 880-889.

56. Galzitskaya, O.V.; Kravchenko, S.V.; Grishin, S.Y.; Zakhareva, A.P.; Mustaeva, L.G.; Gorbunova, E.Y.; Surin, A.K.; Azev, V.N. Combinatorial effects of CPP-modified antimicrobial peptides: Synergistic and additive interactions against pathogenic bacteria. *Int J Mol Sci* **2025**, *26*, 3390.
57. Fields, F.R.; Manzo, G.; Hind, C.K.; Janardhanan, J.; Foik, I.P.; Carmo Silva, P.D.; Balsara, R.D.; Clifford, M.; Vu, H.M.; Ross, J.N., et al. Synthetic antimicrobial peptide tuning permits membrane disruption and interpeptide synergy. *ACS Pharmacol Transl Sci* **2020**, *3*, 418-424.
58. Luna-Ramírez, K.; Sani, M.A.; Silva-Sanchez, J.; Jiménez-Vargas, J.M.; Reyna-Flores, F.; Winkel, K.D.; Wright, C.E.; Possani, L.D.; Separovic, F. Membrane interactions and biological activity of antimicrobial peptides from Australian scorpion. *Biochim Biophys Acta* **2014**, *1838*, 2140-2148.
59. Salama, A.H. Combined action of two synthetic ultrashort antimicrobial peptides exhibiting synergistic effects against clinically significant resistant bacteria. *Vet World* **2024**, *17*, 2725-2730.
60. Shi, J.; Zhang, G.; Wu, H.; Ross, C.; Blecha, F.; Ganz, T. Porcine epithelial beta-defensin 1 is expressed in the dorsal tongue at antimicrobial concentrations. *Infect Immun* **1999**, *67*, 3121-3127.
